# Effect of firearms legislation on suicide and homicide in Canada from 1981 to 2016

**DOI:** 10.1371/journal.pone.0234457

**Published:** 2020-06-18

**Authors:** Caillin Langmann

**Affiliations:** Department of Medicine, Division of Emergency Medicine, McMaster University, Hamilton, Ontario, Canada; Uniformed Services University of the Health Sciences, UNITED STATES

## Abstract

Canada implemented a series of laws regulating firearms including background and psychological screening, licensing, and training in the years 1991, 1994, and 2001. The effects of this legislation on suicide and homicide rates were examined over the years 1981 to 2016. Models were constructed using difference-in-difference analysis of firearms and non firearms death rates from 1981 to 2016. In addition, negative binomial regression was used to test for an association between rates of suicide by Canadian Province and firearms prevalence, using licensing rates as a proxy for prevalence. No associated benefit from firearms legislation on aggregate rates of male suicide was found. In men aged 45 to 59 an associated shift from firearms suicide after 1991 and 1994 to an increase in hanging resulted in overall rate ratios of 0.994 (95%CI, 0.978,1.010) and 0.993 (95%CI, 0.980,1.005) respectively. In men 60 and older a similar effect was seen after 1991, 1994, and 2001, that resulted in rate ratios of 0.989 (95%CI, 0.971,1.008), 0.994 (95%CI, 0.979,1.010), and 1.010 (95%CI, 0.998,1.022) respectively. In females a similar effect was only seen after 1991, rate ratio 0.983 (95%CI, 0.956,1.010). No beneficial association was found between legislation and female or male homicide rates. There was no association found with firearm prevalence rates per province and provincial suicide rates, but an increased association with suicide rates was found with rates of low income, increased unemployment, and the percentage of aboriginals in the population. In conclusion, firearms legislation had no associated beneficial effect on overall suicide and homicide rates. Prevalence of firearms ownership was not associated with suicide rates. Multifaceted strategies to reduce mortality associated with firearms may be required such as steps to reduce youth gang membership and violence, community-based suicide prevention programs, and outreach to groups for which access to care may be a particular issue, such as Aboriginals.

## Introduction

Civilian firearms ownership is relatively common in Canada at an estimated rate of 34.7 firearms per 100 people placing it at seventh highest in the list of 230 countries surveyed in the Small Arms Survey 2017, and death caused by the use of firearms is an important and controversial public health issue [1]. Suicide and homicide predominantly account for the causes of death associated with firearms in Canada [2]. In 2017 there were 266 firearm related homicides in Canada with 52% of firearms homicides related to gang activity and this percentage is gradually increasing [3]. Additionally, the majority of homicide victims (74% or 485 victims) and those accused (87% or 459 accused) were male.

Suicide accounts for approximately 4,000 deaths a year in Canada, a rate of 11 to 12 deaths per 100,000 [4]. Hanging is the most common method of death by suicide accounting for 44% of deaths, whereas 16% of suicides are the result of the use of firearms. Males are much more likely to use firearms in 20% of suicides while females only use firearms in 3%.

The regulation and control of firearms in Canada is primarily the responsibility of the Federal government and as such represents an interesting model to study the effects of gun control legislation as the regulations are applied homogenously across the country. Some exceptions to federal control do exist, however, such as the Province of Quebec, having implemented long gun registration in 2019 [5]. Bill C-51, passed by Canada’s House of Commons in 1977, for the purpose of reducing homicide associated with violent crime, required all firearms purchasers to undergo a criminal record check and obtain a Firearms Acquisition Certificate (FAC) prior to purchasing a firearm. Mandatory minimum sentences and increased penalties were enacted, search and seizure powers granted, new definitions for prohibited and restricted firearms, and individuals were no longer allowed to register handguns at commercial addresses.

The controls enacted under Bill C-51 remained in place for a decade until mounting pressure after a mass homicide at the Polytechnique Institute in Quebec caused Parliament to completely redesign Canadian gun control. In the attempt to reduce all deaths associated with firearms, Canada enacted significant legislation in 1991 (C-17) and 1995 (C-68). C-17, passed in 1991 added two personal reference checks from people familiar with the applicant as well as required spousal endorsement, photo identification, safety training involving written and practical testing, psychological questionnaires, and a mandatory waiting period prior to obtaining a FAC. The psychological questionnaire was designed to screen applicants for a past history of mental health diagnosis associated with an increase risk of suicide or violence. Safe storage laws, transportation laws, magazine capacity restrictions, prohibition of fully automatic firearms, restrictions on military appearing firearms, and new criminal code offences and minimum sentences were also added. Furthermore in 1995, Bill C-68 introduced two types of licenses to replace the FAC, Possession-Only (POL) and Possession and Acquisition (PAL) and added further screening of licensees, a license needed to purchase ammunition, regulated the requirements of authorization to transport restricted firearms such as handguns, and enacted harsher sentences for serious crimes involving firearms. The difference between the previous FAC vs. the PAL/POL is important to note. The FAC was only required at initial time of purchase of a firearm, whereas after the PAL/POL was implemented, possession of a firearm required a valid license, licenses required renewal every 5 years, and holders of PAL/POLs are checked daily to ensure that the holder is not subject to court orders prohibiting the possession of weapons as well as any new criminal charge(s). In this way licenses can be revoked, and firearms confiscated should such issues occur.

It should be noted that portions of Canadian legislation are implemented years after their passage, for example the FAC came into effect in 1979 and the PAL/POL in 2001. The psychological questionnaire was first implemented in 1994. As part of C-68, the registration of all rifles and shotguns was mandatory by 2003, known as the “long gun registry”, while handguns have been registered since 1934. However, in 2012 the Government of Canada repealed the registration of long guns. See S1 Table for a comprehensive list of legislation.

Previous studies have looked at the effects of legislation in Canada and its effects on suicide using 1991 as the point of intervention. Caron examined suicide rates on a native reservation in northern Quebec, Canada, between 1986 to 1996 and found that while legislation enacted in 1991 was associated with a reduction suicide by firearm, hanging and suffocation appeared to replace it as a method, and overall suicide rates increased [6]. Specifically, the rate of suicide by firearm decreased from an average rate of 12.7/100,000 between 1986 to 1991, to a rate of 10.0/100,000 between 1992 to 1996 while the rate by other methods increased from 11.8/100,000 to 16.8/100,000 between the same time periods. Amongst men there was a 31% decrease in suicide by firearm while hanging increased by 53.3%. For women, suicide by firearm dropped by 64% while suicide by hanging increased by 26.9% and suicide by poisoning doubled, from 17.9% to 35.5%.

Another study examined suicide rates in youth between ages 15–19 over the years 1979 to 1999 and found, associated with legislation enacted in 1991, a reduction from 60% to 22% in the percentage of death by suicide by firearm with a compensatory increase in the percentage of suicide by hanging from 20% to 60% [7]. This result suggested a substitution effect had occurred, where suicide by firearm was replaced by suicide by hanging. Additionally, a study on males in Quebec from the years 1981 to 2006 using Joinpoint analysis found that there was a decrease in suicide by firearm that occurred at the same time as firearms legislation was enacted in 1991 [8].

These studies may be subject to error as they examined post legislation trends over only a 5 and 8 year time span or did not control for any potential confounding variables that have shown to be associated with suicide in previous studies, such as poverty rates, percentage of Native American population, unemployment rates, and alcohol use [9,10]. In addition, no previous studies have examined the implementation of psychological screening that was introduced in 1994, and firearms licensing implemented in 2001 in Canada.

The association between firearm legislation and homicide by firearms in Canada has also been examined. The most recent study on overall homicide examined the years 1974 to 2008 and found no associated relationship between homicide rates and firearms legislation enacted in 1977, 1991, and 1995 [11]. Another study looked specifically at homicide of female spouses by firearms and also found no associated benefit with the 1995 legislation, however it did not include any potential explanatory variables such as poverty and unemployment rates [11,12]. A third study examined the legislation enacted in 1991 and found some associated reduction in firearms homicide but this study only examined 7 years pre and post legislation and did not include potential confounders [13].

In this study, trends and levels of firearms homicide and suicide were examined in Canada over the years 1981 to 2016. Since many studies of legislative intervention potentially suffer from errors due to confounding variables, the impact of Canadian legislation was assessed using a difference-in-difference (DiD) approach, a method that can mitigate the potential effects of confounders. This is the first study to look at specific points of firearms legislation implemented after 1991 and the effects on suicide by firearm in Canada as well as the first study to examine homicide in Canada by firearm using a DiD approach.

Since all firearms owners in Canada have been required to hold a license since 2001, it is possible to use that data as a proxy for the availability of firearms per person. It has been hypothesized that increased availability of firearms increases the rate of firearms suicide and therefore overall suicide due to the high lethality of that method [14]. This is the first study to examine the prevalence of firearms and suicide in Canada using licensing as a proxy for availability. A regression model was created to test that relationship.

## Materials and methods

Mortality data was obtained from Statistics Canada [15]. Homicide statistics from the years 1981 to 2016 were obtained from the Canadian Socio-Economic Information Management System (CANSIM) table 35-10-0072-01, 102–0551, 13-10-0156-01, and table 35-10-0069-01. Homicide was defined based on International Classification of Diseases (ICD) codes appropriate to the era (ICD-9 1981–1999: All Assault Homicide E960-E969, Firearms E965; ICD-10 2000–2016: Assault X85-Y09, Y87.1, Firearms X93-X95). Due to confidentiality reasons and internal regulations at Statistics Canada, gender breakdown of homicide data was not obtainable per Province.

Suicide was defined based on International Classification of Diseases codes appropriate to the era (ICD-9 1981–1999: Suicide and Self Inflicted Injury E950-E959, Suicide and Self Inflicted Injury by Hanging, Strangulation and Suffocation E953, Suicide and Self Inflicted Injury by Jumping from a High Place E957, Suicide and Self Inflicted Injury by Firearms E955.0-E955.4; ICD-10 2000–2016: Intentional Self Harm X60-X84, Intentional Self-Harm by Hanging, Strangulation, and Suffocation X70, Intentional Self-Harm by Jumping From a High Place X80, Intentional Self-Harm by Handgun Discharge, Rifle, Shotgun, and Larger Firearm Discharge, and Other and Unspecified Firearm Discharge X72-X74). Population data from the years 1981 to 2016 were obtained from Statistics Canada CANSIM table 051–0001.

Unemployment data was obtained from Statistics Canada table 14-10-0090-01. -Alcohol-volume-purchased-per-capita obtained from Statistics Canada table 10-10-0010-01. Aboriginal population obtained from the Canadian Census 2011 and 2016. Low-income-persons-per-province were obtained from Statistics Canada table 11-10-0018-01. Data for the number of firearms licenses in Canada by region was obtained from the Canadian Firearms Program annual reports [16].

### Statistical analysis

The study was constructed with the null hypothesis that firearms regulations implemented in 1991, 1994, and 2001 were not associated with reductions in the rate of suicide and homicide by firearms. A Difference in differences (DiD) technique was used to construct a quasi experimental time series analysis to compare a control group to a treatment group exposed to the effects of firearms legislation. The benefits of using this model is that it mitigates the effects of external confounders and potential selection bias involved in choosing independent variables to include in regression. Two dependent variables were investigated, suicide and homicide. In the study of suicide, suicide by hanging was used as the control group as it was not expected to be directly affected by firearms legislation, while the treatment group consisted of suicide by firearm. Suicide by hanging was also chosen as the control group, as it is reported to be almost as likely to result in death as suicide by gunshot, and is the most frequent method used by males [14]. Sensitivity tests were performed using all non-firearm and firearm suicide data in the model in order to ensure that a switch from methods other than hanging into hanging, e.g.: a switch from use of poisoning to hanging, was not responsible for any substitution effects. Sensitivity tests were also performed using non-firearm-non-hanging suicide data for men, and suicide by jumping from a high place for women, to test whether any changes in suicide by firearm were independent of changes in hanging. In the study of homicide, the control group consisted of non firearms homicide while the treatment group consisted of homicide by firearms. (Non firearm homicide was calculated as per the ICD era as follows: ICD-9 1981–1999: All Assault Homicide E960-E969 minus Firearms E965; ICD-10 2000–2016: Assault X85-Y09, Y87.1 minus Firearms X93-X95).

A Generalized DiD models was constructed to allow for the relaxation of the parallel trends assumption, in this study a model was constructed including terms to account for differing trends prior to legislation in the control and treatment group as well as changes in trends after legislation in each group [17,18]. Observational quasi experimental designs are also unable to control for crossover from one group to another, in this case while it was expected that firearms legislation would not directly have an effect on suicide by hanging or non firearm homicide, it would potentially be the case that people who were unable to use firearms for suicide or homicide would be forced to choose another method and thus “crossover” into the respective non firearms groups. Constructing a model that includes all pre and post trends can allow for an accounting of the crossover.

The Generalized DiD model was constructed with variables for year (x_i1_), cause of death: firearms or hanging for the analysis of suicide or firearm and non firearm for the analysis of homicide (x_i2_), and a variable to account for whether legislation was in effect (x_i3_). The model utilized the variable “year (x_i1_)” as a term to construct a linear time trend, with interaction terms to allow for different time trends by the cause of death. To account for whether there is a variation in changes in each suicide or homicide category, an interaction between the step term, legislation in effect, and cause of death was included. To allow for a common effect on the trend, an interaction between year and the step term was included. Finally, a 3-way interaction between year, cause of death, and legislation was included and is the difference in difference term that represented the additional effect of legislation. The population of each cohort at that year, n_i_, was used in the model as an offset to ensure changes in population were accounted for. The equation is written as follows:

$$ln\left(Death\;rate\right)=\alpha +ln\left(n_{i}\right)+\beta_{1}x_{i1}+\beta_{2}x_{i2}+\beta_{3}x_{i3}+\beta_{4}x_{i1}x_{i2}+\beta_{5}x_{i2}x_{i3}+\beta_{6}x_{i1}x_{i3}+\beta_{7}x_{i1}x_{i2}x_{i3}$$

The intercept term is indicated by α. The coefficient β_1_ measures the time trend in non firearm mortality before the implementation of legislation, β_2_ measures the rate ratio of mortali in firearm vs. non firearm mortality at the starting year (1981), β_3_ measures the level change in non firearm mortality after the implementation of legislation, β_4_ measures the difference in trend for firearms relative to non firearms before the implementation of legislation, and β_5_ measures the level change in firearm mortality after the implementation of legislation relative to non firearm mortality. The coefficient, β_6,_ measures the change in trend in non firearm mortality after the implementation of legislation. Finally, the 3-way interaction coefficient, β_7,_ measures the additional change in trend in firearm mortality relative to non firearm mortality after legislation, is known as the difference in difference coefficient, and if significant it indicates that the effect of legislation on time trends differed between the non firearm and firearm categories. This 3-way term, β_7_, is the specific measure of the impact of the intervention.

The results were then interpreted as follows, if β_7_ was significant and less than 0 then the firearm mortality trend decreased after the implementation of legislation, while conversely if it is greater than 0 it increased after legislation. If β_6_ and β_7_ were both significant and less than 0 then the non firearm mortality trend decreased after the implementation of legislation, but the firearm mortality trend decreased by a greater amount. If β_6_ and β_7_ were both significant but β_6_ was less than 0 and β_7_ was greater than 0 then the non firearm mortality trend decreased after legislation, but the firearm mortality trend decreased by less or even increased. If β_6_ and β_7_ were both significant and greater than 0, then both non firearm and firearm mortality trends increased after legislation with the firearm mortality trend increasing by a greater amount. If β_6_ and β_7_ were both significant but β_6_ was greater than 0 and β_7_ was less than 0, then the non firearm mortality trend increased after legislation, but the firearm mortality trend decreased or increased by less than the non firearm trend.

Linear combinations of the coefficients were calculated to give an estimate of the annual change in the mortality rate before and after legislation was implemented, and in the case of the DiD coefficient, this was expressed as a rate ratio of the additional rate of firearm mortality post legislation [18]. In addition, the comparisons of post effect trends to account for substitution effects were performed using linear combinations and expressed as rate ratios.

Analysis was conducted over the years 1981 to 2016. 1981 was chosen as a start year as prior legislation came into effect in 1980 and prior years were excluded in order to avoid any effect of these laws. Impacts were set at years 1991, 1994, and 2001 in order to test for the effects of the implementation of each legislation and to conduct sensitivity analysis to account for gradual implementation. Years prior to impacts were coded as 0 and years post impact were coded as 1. The year 1991 was chosen as the implementation of background and reference checks, safe storage regulations, magazine capacity restrictions, mandatory training and the prohibition of a number of firearm types were all implemented at that time. The year 1994 was selected as it was the first year the implementation of the psychological questionnaire was added to background checks. Finally, 2001 was chosen as it was the year that all firearms owners were required to have a firearms license rather than just a certificate to acquire firearms.

A second categorical model was constructed to determine if an association existed between the percentage of the population holding firearms licenses in each Canadian Province or Territory and the rates of suicide by all methods, firearms, and non-firearms methods using negative binomial regression. Suicide rates were examined over the years 2011 to 2016. While licensing in Canada was implemented in 2001, it is estimated that there would be a period of time after implementation where firearms owners would not have obtained a license and that gradual increases in licensing rates would simply be people who already own firearms who have finally obtained a license[19]. Moreover, data on license holders was only sporadically reported by the Canadian Firearms Program and reported during the years up to 2011.

Model 1 was constructed containing a factorial dummy variable for each province or territory to account for intra-provincial effects, a dummy variable for each year, and then a variable for percent license holders. Model 2 contained the variables in Model 1 plus variables for alcohol consumption, unemployment rates, percent aboriginal population, and percentage of low income persons. These variables were examined as they have been included in regression models in previous studies as potentially associated with suicide rates [9,10,20]. The procedure of principal components was used to create a single variable containing the three variables as there existed a high degree of collinearity between variables. The equation is written as follows with population, n_i_, used in the model as an offset to ensure changes in population were accounted, and the intercept term is indicated by α.

$$ln\left(suicide\right)=\alpha +ln\left(n_{i}\right)+\beta_{1}Year+\beta_{2}Province+\beta_{3}Percent\_Licence+\beta_{4}Variable$$

Negative Binomial Regression with standard errors estimated by bootstrapping in Stata/IC version 14 (StataCorp LP, College Station, Texas) was used for statistical analysis. The acceptance level of statistical significance used in the analysis was a p value ss than 0.05 and 95% confidence intervals (CI). False discovery rates were calculated using the Benjamini-Hochberg procedure[21].

## Results

**Fig 1:**
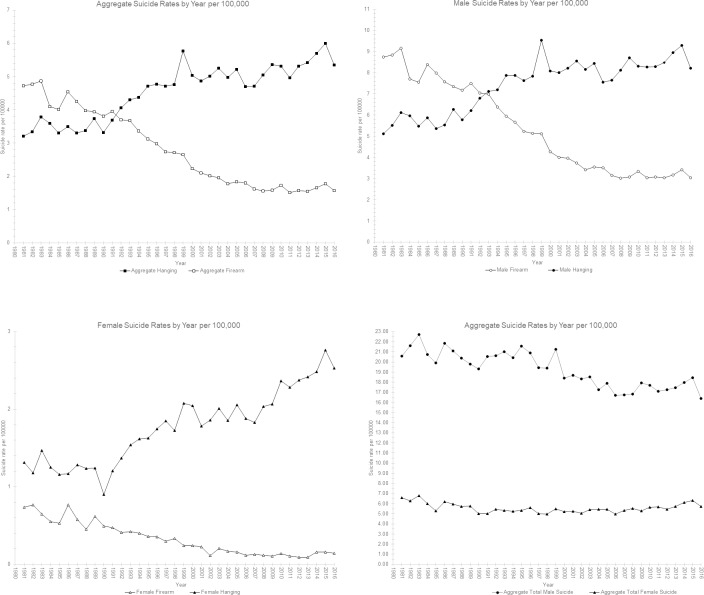
**A–D**: Suicide Rate by Year per 100,000.

Fig 1A–1D display the suicide rates over 1981 to 2016 by aggregate suicide by firearms, aggregate suicide by hanging, total aggregate suicide, as well as by gender. (See S2 Table for suicide numbers and rates from 1981 to 2016 by gender and age brackets). There has been a gradual trend of an increase in suicide by hanging from 1981 onwards with a decrease in suicide by firearm in both sexes. In 1981 the male suicide rate by hanging and firearms were 5.12 per 100,000 and 8.74 per 100,000 respectively, while in 2016 the rates were 8.22 per 100,000 and 3.04 per 100,000 respectively. In females the rates are substantially lower than in males. In 1981 the rates by hanging and firearms were 1.32 per 100,000 and 0.74 per 100,000 respectively, while in 2016 the rates were 2.53 per 100,000 and 0.14 per 100,000 respectively.

**Fig 2:**
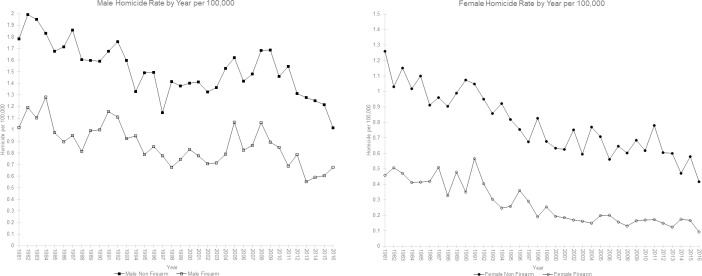
Homicide rate by year per 100,000.

Fig 2A and 2B show the homicide rates over 1981 to 2016 for male and female homicide victims. (See S2 Table for numbers and rates of homicide by year and gender from 1981 to 2016). Both homicide rates by firearm and non-firearm have been declining over time for males and females. In 1981 in males the rate of homicide by firearm and non-firearm were 1.02 per 100,000 and 1.78 per 100,000 respectively, while in 2016 the rates were 0.68 per 100,000 and 1.02 per 100,000 respectively. In 1981 in females the rate of homicide by firearm and non-firearm were 0.46 per 100,000 and 1.26 per 100,000 respectively, while in 2016 the rates were 0.09 per 100,000 and 0.42 per 100,000 respectively. Males are more often the victim of homicide than females.

To examine for associated effects of firearms legislation on suicide the DiD regression analysis was applied to male and female suicide data separately. In addition, male suicide rates were examined in separate age cohorts as it is possible that gun control may affect different age groups, e.g.: younger males may be prevented from acquiring firearms whereas older males may already have firearms and thus be unaffected by certain legislative changes that prevent acquisition. Female suicide rates were not separated into cohorts as many years had cohorts with very small to zero numbers of female suicide by firearm and thus could not be examined by the above method.

Finally, three separate legislative impacts time points were examined, which included changes to background checks and education as well as safe storage regulations enacted in 1991, background checks that involved psychiatric questionnaires implemented in 1994, and licensing implemented in 2001.

Table 1 contains the results from the analysis of suicide rates by hanging or firearm as a rate ratio for the additional change in firearm mortality after legislation as compared to the other rates. A rate ratio of less than 1 suggests that the rate of firearms suicide is declining by more than hanging over time, while a rate ratio greater than 1 suggests that the rate of suicide by firearm is increasing compared to hanging. (See S4 Table for the regression coefficients as well as the percent change per year of hanging and firearms suicide).

**Table 1:** Trend of firearm mortality after legislation.

Age and Gender Cohort by Effect Year	Suicide Rate Ratio^1^	Homicide Rate Ratio^2^
	(95% CI)	(95% CI)
**Aggregate Male**		
**1991 Safe Storage**		
firearm mortality after law^3^	0.987 (0.970, 1.005)	0.989 (0.952, 1.027)
**1994 Psychiatric Questionnaire**		
firearm mortality after law^3^	1.000 (0.984, 1.016)	0.990 (0.962, 1.019)
**2001 Licensing**		
firearm mortality after law^3^	1.040 (1.026, 1.055)	0.997 (0.974, 1.021)
**Male Age 15 to 29**		
**1991 Safe Storage**		
firearm mortality after law^3^	1.005 (0.974, 1.037)	
**1994 Psychiatric Questionnaire**		
firearm mortality after law^3^	1.013 (0.988, 1.038)	
**2001 Licensing**		
firearm mortality after law^3^	1.076 (1.048, 1.105)	
**Male Age 30 to 44**		
**1991 Safe Storage**		
firearm mortality after law^3^	0.984 (0.964, 1.004)	
**1994 Psychiatric Questionnaire**		
firearm mortality after law^3^	1.000 (0.983, 1.022)	
**2001 Licensing**		
firearm mortality after law^3^	1.050 (1.031, 1.069)	
**Male Age 45 to 59**		
**1991 Safe Storage**		
firearm mortality after law^3^	0.945 (0.916, 0.975)	
**1994 Psychiatric Questionnaire**		
firearm mortality after law^3^	0.963 (0.936, 0.991)	
**2001 Licensing**		
firearm mortality after law^3^	1.000 (0.981, 1.019)	
**Male Age 60 plus**		
**1991 Safe Storage**		
firearm mortality after law^3^	0.946 (0.915, 0.978)	
**1994 Psychiatric Questionnaire**		
firearm mortality after law^3^	0.953 (0.931, 0.977)	
**2001 Licensing**		
firearm mortality after law^3^	0.977 (0.959, 0.996)	
**Aggregate Female**		
**1991 Safe Storage**		
firearm mortality after law^3^	0.947 (0.911, 0.984)	0.971 (0.930, 1.014)
**1994 Psychiatric Questionnaire**		
firearm mortality after law^3^	0.974 (0.941, 1.009)	0.983 (0.950, 1.018)
**2001 Licensing**		
firearm mortality after law^3^	1.036 (0.996, 1.077)	1.017 (0.986, 1.048)

^1^The rate ratio of the trend of firearm mortality after each year of legislation implementation which is the difference-in-difference regression result. A rate ratio greater than 1 suggests that the trend of firearm mortality by suicide increased greater than the trend in suicide by hanging while a ratio less than 1 suggests there is a decrease in the trend of suicide by firearm compared to hanging.

^2^The rate ratio of the trend of firearm mortality after each year of legislation implementation which is the difference-in-difference regression result. A rate ratio greater than 1 suggests that the trend of firearm mortality by homicide increased greater than the trend in homicide by other methods while a ratio less than 1 suggests there is a decrease in the trend of homicide by firearm compared to other methods.

^3^Additional change in trend.

Prior to 1991 the trend of aggregate male suicide by hanging was increasing at a very slow rate, [percent change per year was 0.738% (95% CI, -0.494%, 1.994%)] while suicide by firearm was declining [-2.277% (95% CI, -3.238%, -1.325%)]. The trend in aggregate male suicide rates by hanging was not statistically different after 1991 [0.360% (95% CI, 0.001%, 0.719%)] nor was the trend in suicide rates by firearm [-3.981% (95% CI, -4.753%, -3.215%)]. The rate ratio between the change of rate of firearms suicide vs hanging after 1991 was 0.987 (95% CI, 0.970, 1.005) and therefore suggested no benefit of the legislation of firearms on suicide by firearms. These results were similar after the implementation of the 1994 psychiatric questionnaire in that there is no associated beneficial effect on suicide by firearms.

Prior to 2001, aggregate male suicide by hanging was increasing at a rate of 2.453% (95% CI, 1.802%, 3.099%) while aggregate male suicide by firearm decreased at a rate of -3.597% (95% CI, -4.275%, -2.923%). After the implementation of licensing in 2001, the trend of aggregate male suicide rates by hanging decreased to a new rate of -0.284% (95% CI, -0.267%, 0.831%) however the trend of suicide by firearm increased to a new rate of -1.787% (95% CI, -2.672%, -0.909%). The rate ratio between the change of rate of firearms suicide vs hanging after 2001 was 1.040 (95% CI, 1.026, 1.055) and therefore demonstrated an increase in firearms suicide compared to hanging.

For males age 15 to 29 years, the only significant change in trend of suicide by firearms occurred in 2001, and it resulted in an increase in the trend. For males age 30 to 44 years of age a similar result was also found.

In males age 45 to 59 years there is an increase in the regression coefficient indicating the trend of suicide by hanging increased in 1991 and 1994 by 0.051 (95% CI, 0.025, 0.077), and 0.030 (95% CI, 0.004, 0.057), respectively (S4 Table). The additional change in trend in suicide by firearm for those years as indicated by the regression coefficient was -0.057 (95% CI, -0.088, -0.026), and -0.038 (95% CI, -0.067, -0.009). What could be occurring is that as suicide by firearms decreases, suicide by hanging increases by a similar amount. Linear combinations of the regression coefficients was used to test if the parameters were equal and these were expressed as a rate ratio. This resulted in a combined rate ratio of 0.994 (95% CI, 0.978, 1.010) for 1991 and 0.993 (95% CI, 0.980, 1.005) for 1994, suggesting that as the rate of suicide by firearms decreased, an equivalent increase in suicide by hanging occurred. For males aged 60 years and over a similar substitution effect was also seen for years 1991, 1994, and 2001, Table 2.

**Table 2:** Rate ratio of the post effect trends of suicide by hanging and firearms.

Variable	Combination of post effect trend, rate ratio (95% CI)	P
**Suicide**		
Male Age 45 to 59, 1991	0.994 (0.978, 1.010)	0.46
Male Age 45 to 59, 1994	0.993 (0.980, 1.005)	0.25
Male Age 60+, 1991	0.989 (0.971, 1.008)	0.28
Male Age 60+, 1994	0.994 (0.979, 1.010)	0.40
Male Age 60+, 2001	1.010 (0.998, 1.022)	0.09
Female, 1991	0.983 (0.956, 1.010)	0.21

Results of linear combination calculations of post effect rate ratios for hanging and firearms suicide demonstrating method substitution of firearm suicide with hanging. Linear combinations of the addition of the regression coefficients, expressed as rate ratios.

Similar results are shown on Table 1 for aggregate female suicide with no decrease in a change in the trend of suicide after 1994 and 2001. In 1991 an increase in the change in trend of hanging accompanied by a decrease in the change in trend of suicide by firearms resulted in no overall change in suicide rates.

Sensitivity tests were performed using all non-firearm and firearm suicide data in the model to test whether a switch from methods other than hanging into hanging was responsible for the increase in hanging found in the above substitution effect. S5 Table demonstrates similar results to Table 2. This supports the results of a substitution effect from suicide by firearm to hanging in those cohorts and not a switch into hanging from other methods of suicide. A second round of sensitivity tests were performed using non-firearm-non-hanging data for males and suicide by jumping from a high place data for females. For all intervention years, no associated decreased in the rate of suicide by firearm was found in males 45 years and older, and all females, suggesting that suicide by hanging had replaced firearms as a method, and that the trend of suicide by firearm had not changed (S6 Table).

Table 1 reproduces the results from the DiD analysis of homicide by non-firearms and firearms for males and females. Population age cohorts were included in the model to account for variations by age composition. Separate regressions were also conducted for legislative changes in 1991, 1994, and 2001 for both male and female homicide victims. As can be seen, none of the post legislative trends resulted in a decline in firearms homicide in any of the models. (See S7 Table for the regression coefficients as well as the percent change per year of non-firearms and firearms homicide).

**Fig 3:**
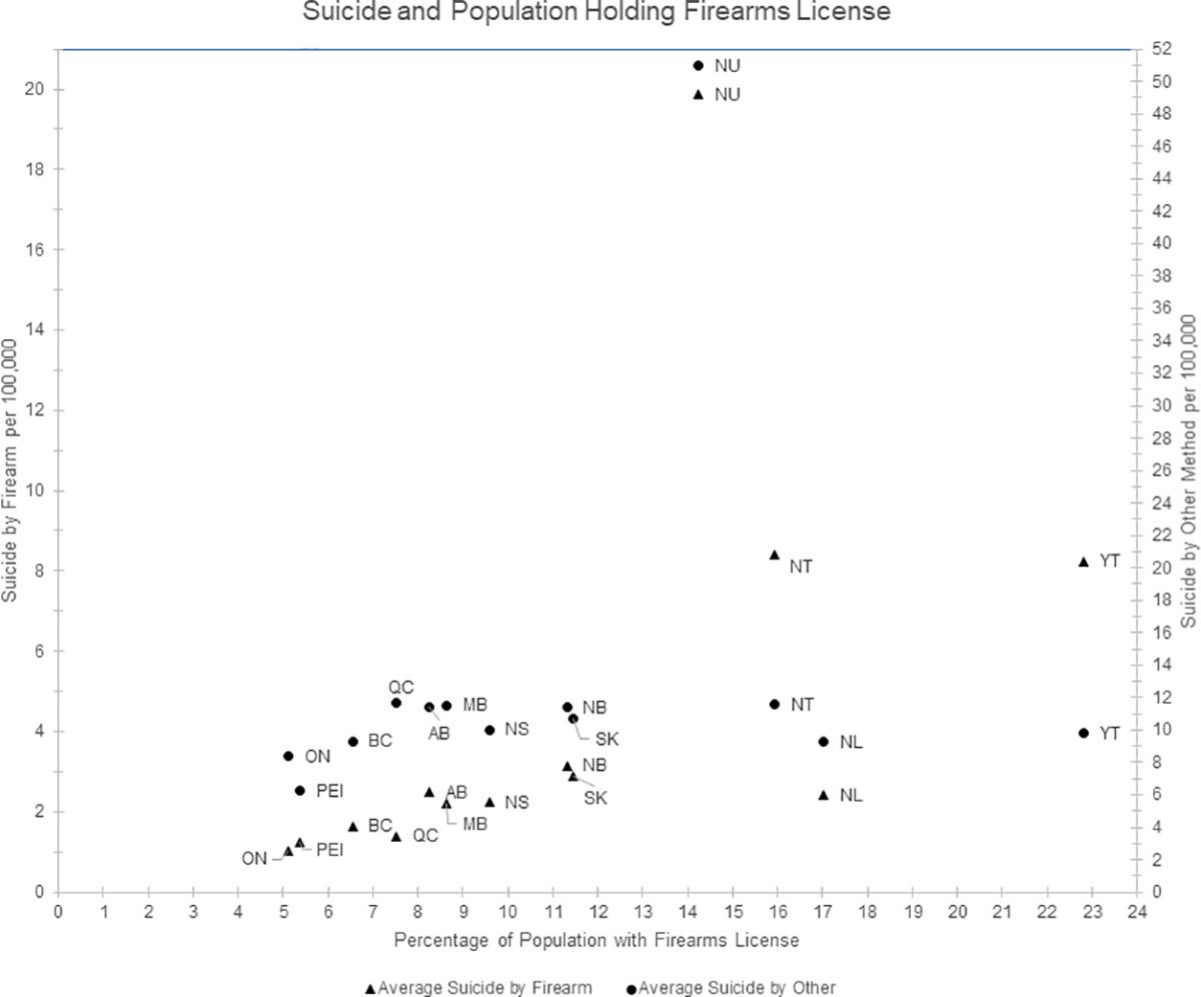
Suicide rate by firearm or other method per Canadian Province or territory by rate of firearms licensing. BC = British Columbia, AB = Alberta, SK = Saskatchewan, MB = Manitoba, ON = Ontario, QC = Quebec, NB = New Brunswick, NS = Nova Scotia, PEI = Prince Edward Island, NL = Newfoundland and Labrador, YT = Yukon, NT = Northwest Territories, NU = Nunuvat.

Table 3 displays the results of the categorical regression model of suicide broken down by province and territory. The suicide rate averaged over 2011 to 2016 per Canadian Province charted against the average rate of firearms licensing per Province is depicted in Fig 3.

**Table 3:** Risk associated between firearm prevalence and suicide rates.

Variable	Univariate	Model 1	Model 2
	Rate Ratio (95% CI)	Rate Ratio (95% CI)	Rate Ratio (95% CI)
**All Suicide**			
Firearms License	-	1.084 (0.975, 1.206)	1.053 (0.941, 1.180)
Regression Factor^a^	1.239 (0.988, 1.554)*	-	1.189 (0.932, 1.517)
Aboriginal Population Rate	1.084 (0.942, 1.247)		
Unemployment Rate	1.029 (0.993, 1.067)*		
Alcohol Volume Per Capita	1.045 (0.933, 1.171)		
Low Income Rate	1.044 (0.988, 1.104)*		
**Suicide by Firearm**			
Firearms License	-	1.092 (0.935, 1.276)	1.031 (0.875, 1.215)
Regression Factor^a^	1.476 (1.043, 2.087)**	-	1.437 (0.987, 2.093)*
Aboriginal Population Rate	1.151 (0.945, 1.401)		
Unemployment Rate	1.050 (0.995, 1.107)*		
Alcohol Volume Per Capita	1.083 (0.901, 1.302)		
Low Income Rate	1.086 (0.995, 1.185)*		
**Suicide by Other Method**			
Firearms License	-	1.081 (0.963, 1.214)	1.057 (0.934, 1.196)
Regression Factor^a^	1.195 (0.936, 1.525)	-	1.145 (0.881, 1.488)
Aboriginal Population Rate	1.067 (0.916, 1.244)		
Unemployment Rate	1.025 (0.986, 1.065)		
Alcohol Volume Per Capita	1.038 (0.921, 1.170)		
Low Income Rate	1.036 (0.977, 1.100)		

Regression results of suicide rates by Province and Territory by Model 1, firearm licensing rates per Province/Territory, and Model 2 including unemployment, aboriginal population rate, and low-income population rate.

* = P ≤ 0.10

** = P ≤ 0.05

^a^ Regression Factor is a principal factor component created from Unemployment, Aboriginal Population, and Percentage of Population Low Income.

Model 1 demonstrates no significant association between the percent number of license holders per population in each province and territory and the rates of suicide by all methods, firearms, and non-firearms. Interestingly, in the univariate analysis and in Model 2, a variable composed of the principal components of three variables: unemployment rates, percent component of aboriginal population of each province or territory, and percentage of the population of each province or territory in low income brackets, demonstrated a positive association with the rates of suicide in each province by all methods and by firearm.

## Discussion

There is considerable debate whether limiting the exposure of people to a highly potent method of suicide, firearms, will decrease overall suicide rates. It is hypothesized that even if people have to switch to another method, the decreased lethality of such methods will result in failed suicide attempts and the possibility of an intervention that will prevent future events [22]. However, hanging, a method that is relatively simple to procure and implement, is shown in some studies to be as lethal as a firearm with an effectiveness of 82% vs firearms effectiveness of 83% [14].

A previous Canadian study examining 7 years pre and post legislation in 1995 showed an associated reduction in suicide that was partially compensated for with an increase in suicide by other methods [13]. However, that study did not break suicide rates down into age cohorts.

This study is the first to examine overall suicide rates over time in Canada and break rates down into cohorts by sex and age. Overall, at the aggregate male level there does not appear to be a reduction in suicide associated with firearms legislation and regulation. Within age cohorts there does appear to be a shift in firearm suicide rates found for males over the age of 45 years to an increase in hanging rates suggesting a complete method substitution effect. Sensitivity tests demonstrating no change in firearm suicide associated with legislation, using non-hanging-non-firearms methods as a control, and jumping from a high place as a control in the female population, suggested that there may be another reason for the shift to suicide by hanging in these cohorts that is unrelated to legislation. Why no effect was seen in the younger aged cohorts may be due to more prevalent firearms ownership in older men as new and more restrictive legislation may have made acquisitions of firearms by new owners more difficult. The finding that suicide by hanging accounted for any deaths saved by potential reductions in suicide by firearms is in agreement with other Canadian studies [6,7]. A recent study on Australian gun control showed similar results in terms of a lack of beneficial association between legislation and suicide after taking into account trends in non firearm related suicide [18].

No associated reduction in homicide with firearm legislation and regulation was found. Studies on Canadian legislation and firearm homicide have produced mixed results, but a larger number have found limited effect [23]. Previous work by Leenaars and Lester found that although legislation enacted in 1977 was associated with a reduction in homicide by firearm, after reanalysis and factoring in social and economic variables the association disappeared [24]. Mauser and Holmes also found no association with this legislation and firearm homicide [25]. Examination of all three legislative efforts by Langmann resulted in no finding of any beneficial association with overall firearm homicide and spousal homicide by firearm, and McPhedran and Mauser found no association between legislation and homicide of female spouses by firearms [11,12]. The results from this study largely confirms the previous work while using a DiD approach to control for confounding variables.

Firearms licensing in Canada provided an opportunity to use that as a proxy for firearms prevalence in each Canadian Province, and while this study found a slightly increasing association between the percentage of firearms license holders per province and territory and firearms suicide, it was not statistically significant at the cut off values used in this study. A previous examination using accidental rates of firearms injury as a proxy for firearm prevalence per Province found an associated increase in suicide by firearm as these rates increased, compensated with a decrease in rates of suicide by other method suggesting a substitution effect [26]. This is the first use of licensing data to examine the relationship between firearms prevalence and suicide in Canada, however many categorical studies demonstrating a relationship have been produced in the United States [23]. For instance, Knopov et al. using survey data as a proxy for prevalence determined an association between firearms prevalence and youth suicide rates after controlling for a number of social economic factors [9]. There is considerable debate about the validity of proxies used for firearm prevalence in the United States and this may affect the validity of the results depending on the measure used [27,28]. It is probable that licensing is a more accurate proxy for firearms prevalence than other methods such as surveys or suicide rates by firearm, as it is a mandatory requirement for owning firearms in Canada.

More notable was that firearms suicide and overall suicide rates were associated with intra-provincial and territorial rates of variables such as unemployment, low income rates, and the rates of aboriginal population. Other studies have shown an association between an increase in suicide rates and an increase in rates of poverty, alcohol consumption, and unemployment [10]. Interestingly, Knopov et al. also found a positive association between suicide rates and the percentage of Aboriginal population per State in the United States [9]. In Canada, suicide rates amongst aboriginal people are 2 to 3 times higher than non-Indigenous Canadians and suicide rates amongst the Inuit in Northern Canada are the highest in the world at 10 times the overall rate for Canada [29].

This study has several limitations. Data for suicide could not be disaggregated by age and Province/Territory due to small numbers, Statistics Canada could not release the data under confidentiality regulations, thus regional associations could not be analysed. Another issue is that firearm suicide and homicide are low base-rate events, and therefore changes in response to specific interventions may not have the statistical power necessary to resolve using regression models. Bias may also result from the misclassification of cause of death by coroners. While it is not possible to determine the number of accidental deaths misclassified as suicide or vice versa, the numbers of accidental deaths by firearms and suffocation per age category are in the low single digit ranges in data obtainable from Statistics Canada.

The multiple regressions performed in this study increased the risk of Type 1 errors, known as the multiple testing error. A false discovery rate of 5%, q-value of 0.05, was calculated for the number of regressions executed. In general, null results were found, however males over the age of 45 did have some associated reduction in suicide with firearms legislation. The multiple number of regressions enhances the possibility this is an error, but the fact that it is conserved in the two oldest age cohorts suggests that there may be an underlying effect in that demographic.

During the years 2001 to 2012 Canada had implemented registration of all firearms, as prior to this registration certificates were only required for handguns and some types of rifles and shotguns. This policy existed temporarily and it is therefore difficult to test this for effect due to the small number of data points as well as there being only 4 years of data post cancellation available. While there was a beneficial association of suicide reduction seen in the 45 and older cohort, this effect may dissipate as years progress if registration rather than the psychiatric questionnaire and licensing was the cause.

## Conclusions

The finding of an association between unemployment, low income rates, the rates of aboriginal population, and provinces with a higher rate of suicide underscores and suggests areas for directed public health and harm reduction programs. No overall mortality reduction, but a shift from suicide by firearm in females and males age 45 and older to hanging, associated with current gun control programs, was found. This suggests that gun control methods to reduce suicide by firearms may have benefits but further actions to reduce suicide by controlling for other methods and suicide prevention programs could lower suicide rates in Canada. No associated reductions in homicide with increasing firearms regulations suggests alternative approaches are necessary to reduce homicide by firearm.

Real action towards reducing the number of firearm deaths is necessary and calls to reduce firearms prevalence in the country have once again become a social and political issue [30,31]. Multifaceted strategies to reduce mortality associated with firearms may be required. Steps to reduce youth gang membership and violence through diversion and educational programs have shown promising results [32]. As well community based suicide prevention programs such as training of family physicians in the detection and treatment of depression and discussions about firearms, campaigns aimed at increasing awareness about depression, and follow-up of individuals who attempted suicide may result in lives saved [33]. Outreach to groups for which access to care may be a particular issue, such as Aboriginals, is of primary concern [34].

## Supporting information

S1 Table Timeline and description of Canadian firearms legislation. Year legislation enacted and implemented as well as a brief description of legislation and regulations.
(DOCX)

S2 Table Suicide by method by year. (DOCX)

S3 Table Homicide by method by year. (DOCX)

S4 Table Suicide difference-in-differences results. Results of regression model are expressed as regression coefficients, percentage change per year of suicide rates.
(DOCX)

S5 Table Rate ratio of the post effect trends of suicide by non-firearm and firearms. Results of linear combination calculations of post effect rate ratios for non-firearm and firearms suicide demonstrating method substitution of firearm suicide with hanging. Linear combinations of the addition of the regression coefficients, if statistically equal, should result in a rate ratio of ~1.0 with confidence intervals crossing 1.0.
(DOCX)

S6 Table Trend of suicide by firearm, suicide by non hanging non firearm, and suicide by jumping after intervention. Results of sensitivity tests performed using non hanging non firearm data for males and suicide by jumping data for females. No associated decreased in the rate of suicide by firearm was found in males 45 years and older and all females after 1991 suggesting that suicide by hanging had replaced firearms as a method in the cohorts and interventions where an effect was found in the DiD model. ^1^The rate ratio of the trend of firearm mortality after each year of legislation implementation which is the difference-in-difference regression result. A rate ratio greater than 1 suggests that firearm mortality by suicide or homicide is increasing greater than mortality by other methods, while a ratio less than 1 suggests there is a decrease greater than other methods. ^2^Additional change in trend.
(DOCX)

S7 Table Homicide difference-in-differences results. Results of regression model are expressed as regression coefficients, percentage change per year of homicide rates.
(DOCX)
