# Supplementary material for: Effect of firearms legislation on suicide and homicide in Canada from 1981 to 2016
Source: PLoS One. 2020 Jun 18;15(6):e0234457. doi: 10.1371/journal.pone.0234457 (PMC7302582; doi:10.1371/journal.pone.0234457)
Supplement: S1 Table — Year legislation enacted and implemented as well as a brief description of legislation and regulations. (DOCX) [file pone.0234457.s001.docx]

| **Legislation Enacted/Implemented** | **Effects of Legislation** |
| --- | --- |
| 1977 Bill C51 | - All firearms purchasers to undergo a criminal record check and obtain a Firearms Acquisition Certificate (FAC). - Mandatory minimum sentences and increased penalties - Search and seizure powers granted - New definitions for prohibited and restricted firearms - Individuals no longer allowed to register handguns at commercial addresses. |
| 1979 | - Implementation of Firearms Acquisition Certificate |
| 1991 Bill C-17 | - Two reference checks - Spousal endorsement - Photo identification - Safety training involving written and practical testing - Psychological questionnaires - Mandatory waiting period - Safe storage laws - Transportation laws - Magazine capacity restrictions - Prohibition of fully automatic firearms - Restrictions on military appearing firearms - New criminal code offences - Minimum sentences |
| 1994 | - Implementation of Psychological Questionnaire |
| 1995 Bill C-68 | - Two types of licenses in place of the FAC, Possession-Only (POL) and Possession and Acquisition (PAL) - Further screening of licensees - License needed to purchase ammunition - Requirements of authorization to transport restricted firearms - Increased sentences for serious crimes involving firearms. |
| 2001 | - Implementation of the POL/PAL License system |
| 2003 | - Implementation of mandatory registration of all rifles and shotguns |
| 2012 | - Repeal of the registration of rifles and shotguns |
