# Supplementary material for: Effect of firearms legislation on suicide and homicide in Canada from 1981 to 2016
Source: PLoS One. 2020 Jun 18;15(6):e0234457. doi: 10.1371/journal.pone.0234457 (PMC7302582; doi:10.1371/journal.pone.0234457)
Supplement: S2 Table — (DOCX) [file pone.0234457.s002.docx]

| **Year** |  | **Total Suicide** | **Hanging** | **Firearm** | **Population** | **Total Suicide per 100000** | **Hanging per 100000** | **Firearm per 100000** |
| --- | --- | --- | --- | --- | --- | --- | --- | --- |
|  |  |  |  |  |  |  |  |  |
| **Male Suicide** | | |  |  |  |  |  |  |
| 1981 |  | 2543 | 633 | 1080 | 12351233 | 20.59 | 5.12 | 8.74 |
| 1982 |  | 2698 | 689 | 1105 | 12490701 | 21.60 | 5.52 | 8.85 |
| 1983 |  | 2865 | 772 | 1155 | 12606769 | 22.73 | 6.12 | 9.16 |
| 1984 |  | 2637 | 759 | 981 | 12717823 | 20.73 | 5.97 | 7.71 |
| 1985 |  | 2552 | 703 | 970 | 12828106 | 19.89 | 5.48 | 7.56 |
| 1986 |  | 2830 | 760 | 1086 | 12951377 | 21.85 | 5.87 | 8.39 |
| 1987 |  | 2767 | 704 | 1049 | 13125126 | 21.08 | 5.36 | 7.99 |
| 1988 |  | 2710 | 736 | 1007 | 13289039 | 20.39 | 5.54 | 7.58 |
| 1989 |  | 2676 | 847 | 994 | 13524501 | 19.79 | 6.26 | 7.35 |
| 1990 |  | 2650 | 793 | 985 | 13721733 | 19.31 | 5.78 | 7.18 |
| 1991 |  | 2857 | 864 | 1043 | 13904391 | 20.55 | 6.21 | 7.50 |
| 1992 |  | 2896 | 955 | 991 | 14055134 | 20.60 | 6.79 | 7.05 |
| 1993 |  | 2987 | 1011 | 993 | 14205297 | 21.03 | 7.12 | 6.99 |
| 1994 |  | 2931 | 1033 | 916 | 14357184 | 20.41 | 7.20 | 6.38 |
| 1995 |  | 3125 | 1141 | 863 | 14502481 | 21.55 | 7.87 | 5.95 |
| 1996 |  | 3061 | 1153 | 830 | 14650314 | 20.89 | 7.87 | 5.67 |
| 1997 |  | 2875 | 1130 | 773 | 14806454 | 19.42 | 7.63 | 5.22 |
| 1998 |  | 2895 | 1171 | 767 | 14925127 | 19.40 | 7.85 | 5.14 |
| 1999 |  | 3199 | 1436 | 770 | 15048669 | 21.26 | 9.54 | 5.12 |
| 2000 |  | 2798 | 1229 | 648 | 15193812 | 18.42 | 8.09 | 4.26 |
| 2001 |  | 2869 | 1230 | 616 | 15366600 | 18.67 | 8.00 | 4.01 |
| 2002 |  | 2849 | 1276 | 615 | 15537576 | 18.34 | 8.21 | 3.96 |
| 2003 |  | 2902 | 1341 | 585 | 15679310 | 18.51 | 8.55 | 3.73 |
| 2004 |  | 2734 | 1291 | 541 | 15827090 | 17.27 | 8.16 | 3.42 |
| 2005 |  | 2856 | 1348 | 567 | 15980008 | 17.87 | 8.44 | 3.55 |
| 2006 |  | 2695 | 1220 | 567 | 16144759 | 16.69 | 7.56 | 3.51 |
| 2007 |  | 2726 | 1248 | 513 | 16298852 | 16.73 | 7.66 | 3.15 |
| 2008 |  | 2773 | 1337 | 498 | 16474178 | 16.83 | 8.12 | 3.02 |
| 2009 |  | 2986 | 1451 | 513 | 16663413 | 17.92 | 8.71 | 3.08 |
| 2010 |  | 2980 | 1399 | 563 | 16847823 | 17.69 | 8.30 | 3.34 |
| 2011 |  | 2909 | 1408 | 518 | 17014528 | 17.10 | 8.28 | 3.04 |
| 2012 |  | 2970 | 1427 | 528 | 17209900 | 17.26 | 8.29 | 3.07 |
| 2013 |  | 3038 | 1476 | 528 | 17401165 | 17.46 | 8.48 | 3.03 |
| 2014 |  | 3156 | 1574 | 559 | 17581697 | 17.95 | 8.95 | 3.18 |
| 2015 |  | 3266 | 1646 | 604 | 17712801 | 18.44 | 9.29 | 3.41 |
| 2016 |  | 2935 | 1473 | 544 | 17916496 | 16.38 | 8.22 | 3.04 |
|  |  |  |  |  |  |  |  |  |
| **Male Suicide Age 15 - 29** | | | |  |  |  |  |  |
| 1981 |  | 916 | 214 | 444 | 3590526 | 25.51 | 5.96 | 12.37 |
| 1982 |  | 996 | 259 | 443 | 3599743 | 27.67 | 7.19 | 12.31 |
| 1983 |  | 1042 | 293 | 459 | 3587781 | 29.04 | 8.17 | 12.79 |
| 1984 |  | 936 | 299 | 348 | 3565922 | 26.25 | 8.38 | 9.76 |
| 1985 |  | 881 | 236 | 363 | 3540298 | 24.88 | 6.67 | 10.25 |
| 1986 |  | 956 | 280 | 387 | 3522493 | 27.14 | 7.95 | 10.99 |
| 1987 |  | 899 | 244 | 361 | 3488732 | 25.77 | 6.99 | 10.35 |
| 1988 |  | 906 | 304 | 329 | 3440643 | 26.33 | 8.84 | 9.56 |
| 1989 |  | 866 | 307 | 334 | 3417831 | 25.34 | 8.98 | 9.77 |
| 1990 |  | 831 | 281 | 334 | 3379013 | 24.59 | 8.32 | 9.88 |
| 1991 |  | 890 | 344 | 324 | 3325441 | 26.76 | 10.34 | 9.74 |
| 1992 |  | 865 | 323 | 344 | 3272557 | 26.43 | 9.87 | 10.51 |
| 1993 |  | 822 | 330 | 283 | 3215866 | 25.56 | 10.26 | 8.80 |
| 1994 |  | 789 | 330 | 234 | 3172531 | 24.87 | 10.40 | 7.38 |
| 1995 |  | 832 | 368 | 208 | 3141811 | 26.48 | 11.71 | 6.62 |
| 1996 |  | 779 | 386 | 183 | 3136447 | 24.84 | 12.31 | 5.83 |
| 1997 |  | 682 | 336 | 163 | 3142936 | 21.70 | 10.69 | 5.19 |
| 1998 |  | 721 | 365 | 172 | 3146699 | 22.91 | 11.60 | 5.47 |
| 1999 |  | 733 | 403 | 154 | 3163302 | 23.17 | 12.74 | 4.87 |
| 2000 |  | 648 | 359 | 117 | 3187086 | 20.33 | 11.26 | 3.67 |
| 2001 |  | 638 | 356 | 107 | 3219931 | 19.81 | 11.06 | 3.32 |
| 2002 |  | 600 | 332 | 112 | 3247320 | 18.48 | 10.22 | 3.45 |
| 2003 |  | 606 | 366 | 92 | 3271688 | 18.52 | 11.19 | 2.81 |
| 2004 |  | 597 | 385 | 83 | 3307174 | 18.05 | 11.64 | 2.51 |
| 2005 |  | 573 | 353 | 90 | 3347179 | 17.12 | 10.55 | 2.69 |
| 2006 |  | 506 | 313 | 67 | 3388630 | 14.93 | 9.24 | 1.98 |
| 2007 |  | 582 | 335 | 93 | 3417289 | 17.03 | 9.80 | 2.72 |
| 2008 |  | 537 | 332 | 67 | 3447550 | 15.58 | 9.63 | 1.94 |
| 2009 |  | 550 | 349 | 67 | 3483030 | 15.79 | 10.02 | 1.92 |
| 2010 |  | 563 | 327 | 87 | 3513410 | 16.02 | 9.31 | 2.48 |
| 2011 |  | 580 | 347 | 92 | 3527486 | 16.44 | 9.84 | 2.61 |
| 2012 |  | 598 | 353 | 68 | 3551816 | 16.84 | 9.94 | 1.91 |
| 2013 |  | 514 | 324 | 64 | 3563169 | 14.43 | 9.09 | 1.80 |
| 2014 |  | 570 | 347 | 79 | 3572741 | 15.95 | 9.71 | 2.21 |
| 2015 |  | 629 | 382 | 94 | 3564344 | 17.65 | 10.72 | 2.64 |
| 2016 |  | 562 | 342 | 85 | 3581201 | 15.69 | 9.55 | 2.37 |
|  |  |  |  |  |  |  |  |  |
| **Male Suicide Age 30 - 44** | | | |  |  |  |  |  |
| 1981 |  | 667 | 152 | 254 | 2597934 | 25.67 | 5.85 | 9.78 |
| 1982 |  | 691 | 154 | 268 | 2681570 | 25.77 | 5.74 | 9.99 |
| 1983 |  | 751 | 190 | 255 | 2761942 | 27.19 | 6.88 | 9.23 |
| 1984 |  | 719 | 183 | 244 | 2845610 | 25.27 | 6.43 | 8.57 |
| 1985 |  | 722 | 190 | 245 | 2937478 | 24.58 | 6.47 | 8.34 |
| 1986 |  | 853 | 225 | 307 | 3029115 | 28.16 | 7.43 | 10.13 |
| 1987 |  | 825 | 204 | 275 | 3134110 | 26.32 | 6.51 | 8.77 |
| 1988 |  | 828 | 194 | 292 | 3230342 | 25.63 | 6.01 | 9.04 |
| 1989 |  | 843 | 263 | 269 | 3346790 | 25.19 | 7.86 | 8.04 |
| 1990 |  | 910 | 262 | 294 | 3447023 | 26.40 | 7.60 | 8.53 |
| 1991 |  | 947 | 266 | 300 | 3545849 | 26.71 | 7.50 | 8.46 |
| 1992 |  | 1003 | 326 | 283 | 3590440 | 27.94 | 9.08 | 7.88 |
| 1993 |  | 1057 | 368 | 294 | 3651657 | 28.95 | 10.08 | 8.05 |
| 1994 |  | 1050 | 368 | 280 | 3712163 | 28.29 | 9.91 | 7.54 |
| 1995 |  | 1152 | 436 | 264 | 3764089 | 30.61 | 11.58 | 7.01 |
| 1996 |  | 1101 | 406 | 244 | 3792039 | 29.03 | 10.71 | 6.43 |
| 1997 |  | 1010 | 396 | 213 | 3814035 | 26.48 | 10.38 | 5.58 |
| 1998 |  | 1017 | 403 | 214 | 3808143 | 26.71 | 10.58 | 5.62 |
| 1999 |  | 1180 | 535 | 228 | 3797910 | 31.07 | 14.09 | 6.00 |
| 2000 |  | 997 | 458 | 176 | 3790571 | 26.30 | 12.08 | 4.64 |
| 2001 |  | 967 | 428 | 176 | 3791473 | 25.50 | 11.29 | 4.64 |
| 2002 |  | 902 | 451 | 138 | 3778970 | 23.87 | 11.93 | 3.65 |
| 2003 |  | 929 | 482 | 131 | 3745294 | 24.80 | 12.87 | 3.50 |
| 2004 |  | 860 | 439 | 124 | 3709502 | 23.18 | 11.83 | 3.34 |
| 2005 |  | 891 | 451 | 143 | 3675490 | 24.24 | 12.27 | 3.89 |
| 2006 |  | 773 | 371 | 124 | 3635168 | 21.26 | 10.21 | 3.41 |
| 2007 |  | 729 | 368 | 97 | 3589602 | 20.31 | 10.25 | 2.70 |
| 2008 |  | 796 | 414 | 91 | 3545698 | 22.45 | 11.68 | 2.57 |
| 2009 |  | 822 | 435 | 100 | 3510076 | 23.42 | 12.39 | 2.85 |
| 2010 |  | 750 | 387 | 108 | 3488038 | 21.50 | 11.10 | 3.10 |
| 2011 |  | 742 | 387 | 108 | 3489196 | 21.27 | 11.09 | 3.10 |
| 2012 |  | 722 | 381 | 103 | 3512144 | 20.56 | 10.85 | 2.93 |
| 2013 |  | 717 | 403 | 85 | 3539920 | 20.25 | 11.38 | 2.40 |
| 2014 |  | 754 | 427 | 96 | 3564746 | 21.15 | 11.98 | 2.69 |
| 2015 |  | 727 | 418 | 102 | 3571915 | 20.35 | 11.70 | 2.86 |
| 2016 |  | 723 | 398 | 91 | 3603146 | 20.07 | 11.05 | 2.53 |
|  |  |  |  |  |  |  |  |  |
| **Male Suicide Age 45 - 59** | | | |  |  |  |  |  |
| 1981 |  | 533 | 138 | 208 | 1841548 | 28.94 | 7.49 | 11.29 |
| 1982 |  | 582 | 147 | 215 | 1849638 | 31.47 | 7.95 | 11.62 |
| 1983 |  | 556 | 117 | 231 | 1860146 | 29.89 | 6.29 | 12.42 |
| 1984 |  | 547 | 145 | 204 | 1872126 | 29.22 | 7.75 | 10.90 |
| 1985 |  | 501 | 119 | 198 | 1884542 | 26.58 | 6.31 | 10.51 |
| 1986 |  | 528 | 120 | 198 | 1904853 | 27.72 | 6.30 | 10.39 |
| 1987 |  | 566 | 129 | 214 | 1936909 | 29.22 | 6.66 | 11.05 |
| 1988 |  | 494 | 92 | 186 | 1980008 | 24.95 | 4.65 | 9.39 |
| 1989 |  | 501 | 132 | 209 | 2031173 | 24.67 | 6.50 | 10.29 |
| 1990 |  | 478 | 116 | 172 | 2077868 | 23.00 | 5.58 | 8.28 |
| 1991 |  | 533 | 135 | 194 | 2136051 | 24.95 | 6.32 | 9.08 |
| 1992 |  | 573 | 169 | 184 | 2212986 | 25.89 | 7.64 | 8.31 |
| 1993 |  | 632 | 170 | 217 | 2293006 | 27.56 | 7.41 | 9.46 |
| 1994 |  | 602 | 187 | 197 | 2376586 | 25.33 | 7.87 | 8.29 |
| 1995 |  | 676 | 198 | 207 | 2460748 | 27.47 | 8.05 | 8.41 |
| 1996 |  | 662 | 190 | 204 | 2546806 | 25.99 | 7.46 | 8.01 |
| 1997 |  | 708 | 233 | 202 | 2642066 | 26.80 | 8.82 | 7.65 |
| 1998 |  | 676 | 220 | 197 | 2737097 | 24.70 | 8.04 | 7.20 |
| 1999 |  | 795 | 322 | 196 | 2835945 | 28.03 | 11.35 | 6.91 |
| 2000 |  | 687 | 261 | 182 | 2941474 | 23.36 | 8.87 | 6.19 |
| 2001 |  | 831 | 312 | 185 | 3044787 | 27.29 | 10.25 | 6.08 |
| 2002 |  | 842 | 325 | 190 | 3154155 | 26.69 | 10.30 | 6.02 |
| 2003 |  | 848 | 321 | 199 | 3255062 | 26.05 | 9.86 | 6.11 |
| 2004 |  | 815 | 320 | 187 | 3352586 | 24.31 | 9.54 | 5.58 |
| 2005 |  | 890 | 361 | 177 | 3454482 | 25.76 | 10.45 | 5.12 |
| 2006 |  | 869 | 352 | 181 | 3553157 | 24.46 | 9.91 | 5.09 |
| 2007 |  | 899 | 362 | 168 | 3628973 | 24.77 | 9.98 | 4.63 |
| 2008 |  | 882 | 390 | 163 | 3713237 | 23.75 | 10.50 | 4.39 |
| 2009 |  | 1023 | 459 | 166 | 3793812 | 26.96 | 12.10 | 4.38 |
| 2010 |  | 1034 | 458 | 187 | 3857730 | 26.80 | 11.87 | 4.85 |
| 2011 |  | 959 | 450 | 141 | 3893074 | 24.63 | 11.56 | 3.62 |
| 2012 |  | 1007 | 458 | 178 | 3914690 | 25.72 | 11.70 | 4.55 |
| 2013 |  | 1065 | 501 | 181 | 3925677 | 27.13 | 12.76 | 4.61 |
| 2014 |  | 1093 | 525 | 180 | 3925632 | 27.84 | 13.37 | 4.59 |
| 2015 |  | 1074 | 550 | 160 | 3913666 | 27.44 | 14.05 | 4.09 |
| 2016 |  | 933 | 463 | 163 | 3902223 | 23.91 | 11.87 | 4.18 |
|  |  |  |  |  |  |  |  |  |
| **Male Suicide Age 60+** | | | |  |  |  |  |  |
| 1981 |  | 427 | 112 | 165 | 1463342 | 29.18 | 7.65 | 11.28 |
| 1982 |  | 429 | 145 | 170 | 1505574 | 28.49 | 9.63 | 11.29 |
| 1983 |  | 516 | 159 | 204 | 1543516 | 33.43 | 10.30 | 13.22 |
| 1984 |  | 435 | 117 | 178 | 1583861 | 27.46 | 7.39 | 11.24 |
| 1985 |  | 448 | 148 | 161 | 1622956 | 27.60 | 9.12 | 9.92 |
| 1986 |  | 493 | 121 | 188 | 1662996 | 29.65 | 7.28 | 11.30 |
| 1987 |  | 477 | 111 | 190 | 1711926 | 27.86 | 6.48 | 11.10 |
| 1988 |  | 482 | 131 | 193 | 1758610 | 27.41 | 7.45 | 10.97 |
| 1989 |  | 466 | 133 | 175 | 1806473 | 25.80 | 7.36 | 9.69 |
| 1990 |  | 431 | 121 | 175 | 1856291 | 23.22 | 6.52 | 9.43 |
| 1991 |  | 487 | 111 | 214 | 1902476 | 25.60 | 5.83 | 11.25 |
| 1992 |  | 455 | 121 | 173 | 1943213 | 23.41 | 6.23 | 8.90 |
| 1993 |  | 476 | 123 | 192 | 1979889 | 24.04 | 6.21 | 9.70 |
| 1994 |  | 490 | 125 | 194 | 2012475 | 24.35 | 6.21 | 9.64 |
| 1995 |  | 465 | 119 | 182 | 2045015 | 22.74 | 5.82 | 8.90 |
| 1996 |  | 519 | 149 | 193 | 2078257 | 24.97 | 7.17 | 9.29 |
| 1997 |  | 475 | 140 | 181 | 2112930 | 22.48 | 6.63 | 8.57 |
| 1998 |  | 481 | 149 | 176 | 2149019 | 22.38 | 6.93 | 8.19 |
| 1999 |  | 491 | 163 | 181 | 2187605 | 22.44 | 7.45 | 8.27 |
| 2000 |  | 437 | 129 | 168 | 2229605 | 19.60 | 5.79 | 7.53 |
| 2001 |  | 419 | 122 | 146 | 2314699 | 18.10 | 5.27 | 6.31 |
| 2002 |  | 487 | 156 | 171 | 2372938 | 20.52 | 6.57 | 7.21 |
| 2003 |  | 500 | 154 | 162 | 2440303 | 20.49 | 6.31 | 6.64 |
| 2004 |  | 445 | 136 | 142 | 2511288 | 17.72 | 5.42 | 5.65 |
| 2005 |  | 483 | 164 | 157 | 2583470 | 18.70 | 6.35 | 6.08 |
| 2006 |  | 534 | 174 | 192 | 2671991 | 19.99 | 6.51 | 7.19 |
| 2007 |  | 498 | 168 | 154 | 2779084 | 17.92 | 6.05 | 5.54 |
| 2008 |  | 542 | 187 | 176 | 2885661 | 18.78 | 6.48 | 6.10 |
| 2009 |  | 578 | 199 | 176 | 2991774 | 19.32 | 6.65 | 5.88 |
| 2010 |  | 614 | 213 | 178 | 3103360 | 19.79 | 6.86 | 5.74 |
| 2011 |  | 613 | 213 | 174 | 3216380 | 19.06 | 6.62 | 5.41 |
| 2012 |  | 626 | 223 | 176 | 3330165 | 18.80 | 6.70 | 5.29 |
| 2013 |  | 727 | 237 | 195 | 3451320 | 21.06 | 6.87 | 5.65 |
| 2014 |  | 728 | 266 | 202 | 3577055 | 20.35 | 7.44 | 5.65 |
| 2015 |  | 822 | 285 | 245 | 3704158 | 22.19 | 7.69 | 6.61 |
| 2016 |  | 701 | 255 | 204 | 3837842 | 18.27 | 6.64 | 5.32 |
|  |  |  |  |  |  |  |  |  |
| **Female Suicide** | | | |  |  |  |  |  |
| 1981 |  | 822 | 164 | 92 | 12468682 | 6.59 | 1.32 | 0.74 |
| 1982 |  | 791 | 149 | 97 | 12626241 | 6.26 | 1.18 | 0.77 |
| 1983 |  | 867 | 187 | 83 | 12759682 | 6.79 | 1.47 | 0.65 |
| 1984 |  | 774 | 161 | 71 | 12889230 | 6.01 | 1.25 | 0.55 |
| 1985 |  | 688 | 151 | 69 | 13014010 | 5.29 | 1.16 | 0.53 |
| 1986 |  | 815 | 154 | 101 | 13148901 | 6.20 | 1.17 | 0.77 |
| 1987 |  | 794 | 171 | 77 | 13321475 | 5.96 | 1.28 | 0.58 |
| 1988 |  | 772 | 167 | 61 | 13502708 | 5.72 | 1.24 | 0.45 |
| 1989 |  | 790 | 171 | 85 | 13752280 | 5.74 | 1.24 | 0.62 |
| 1990 |  | 698 | 126 | 69 | 13969405 | 5.00 | 0.90 | 0.49 |
| 1991 |  | 709 | 170 | 67 | 14133029 | 5.02 | 1.20 | 0.47 |
| 1992 |  | 778 | 196 | 59 | 14316130 | 5.43 | 1.37 | 0.41 |
| 1993 |  | 771 | 223 | 61 | 14479467 | 5.32 | 1.54 | 0.42 |
| 1994 |  | 768 | 237 | 59 | 14643479 | 5.24 | 1.62 | 0.40 |
| 1995 |  | 791 | 241 | 53 | 14799830 | 5.34 | 1.63 | 0.36 |
| 1996 |  | 839 | 261 | 53 | 14959904 | 5.61 | 1.74 | 0.35 |
| 1997 |  | 755 | 279 | 45 | 15099494 | 5.00 | 1.85 | 0.30 |
| 1998 |  | 758 | 263 | 51 | 15230046 | 4.98 | 1.73 | 0.33 |
| 1999 |  | 843 | 319 | 37 | 15352617 | 5.49 | 2.08 | 0.24 |
| 2000 |  | 807 | 317 | 37 | 15491918 | 5.21 | 2.05 | 0.24 |
| 2001 |  | 819 | 279 | 35 | 15654302 | 5.23 | 1.78 | 0.22 |
| 2002 |  | 799 | 294 | 18 | 15822503 | 5.05 | 1.86 | 0.11 |
| 2003 |  | 862 | 321 | 33 | 15964718 | 5.40 | 2.01 | 0.21 |
| 2004 |  | 879 | 299 | 27 | 16113565 | 5.46 | 1.86 | 0.17 |
| 2005 |  | 885 | 334 | 26 | 16263745 | 5.44 | 2.05 | 0.16 |
| 2006 |  | 816 | 309 | 19 | 16426415 | 4.97 | 1.88 | 0.12 |
| 2007 |  | 882 | 303 | 21 | 16590173 | 5.32 | 1.83 | 0.13 |
| 2008 |  | 927 | 341 | 20 | 16772940 | 5.53 | 2.03 | 0.12 |
| 2009 |  | 899 | 350 | 18 | 16965482 | 5.30 | 2.06 | 0.11 |
| 2010 |  | 968 | 406 | 24 | 17157066 | 5.64 | 2.37 | 0.14 |
| 2011 |  | 985 | 395 | 19 | 17324800 | 5.69 | 2.28 | 0.11 |
| 2012 |  | 954 | 416 | 16 | 17504322 | 5.45 | 2.38 | 0.09 |
| 2013 |  | 1012 | 427 | 16 | 17681789 | 5.72 | 2.41 | 0.09 |
| 2014 |  | 1094 | 443 | 28 | 17855738 | 6.13 | 2.48 | 0.16 |
| 2015 |  | 1136 | 497 | 29 | 17990107 | 6.31 | 2.76 | 0.16 |
| 2016 |  | 1039 | 460 | 26 | 18192991 | 5.71 | 2.53 | 0.14 |
