# Supplementary material for: Effect of firearms legislation on suicide and homicide in Canada from 1981 to 2016
Source: PLoS One. 2020 Jun 18;15(6):e0234457. doi: 10.1371/journal.pone.0234457 (PMC7302582; doi:10.1371/journal.pone.0234457)
Supplement: S3 Table — (DOCX) [file pone.0234457.s003.docx]

| **Year** |  | **Total Homicide** | **Non Firearm** | **Firearm** | **Population** | **Homicide Non Firearm per 100000** | **Homicide Firearm per 100000** |
| --- | --- | --- | --- | --- | --- | --- | --- |
|  |  |  |  |  |  |  |  |
| **Male Homicide** | | |  |  |  |  |  |
| 1981 |  | 346 | 220 | 126 | 12351233 | 1.78 | 1.02 |
| 1982 |  | 398 | 249 | 149 | 12490701 | 1.99 | 1.19 |
| 1983 |  | 385 | 246 | 139 | 12606769 | 1.95 | 1.10 |
| 1984 |  | 396 | 233 | 163 | 12717823 | 1.83 | 1.28 |
| 1985 |  | 340 | 215 | 125 | 12828106 | 1.68 | 0.97 |
| 1986 |  | 338 | 222 | 116 | 12951377 | 1.71 | 0.90 |
| 1987 |  | 369 | 244 | 125 | 13125126 | 1.86 | 0.95 |
| 1988 |  | 321 | 213 | 108 | 13289039 | 1.60 | 0.81 |
| 1989 |  | 350 | 216 | 134 | 13524501 | 1.60 | 0.99 |
| 1990 |  | 355 | 218 | 137 | 13721733 | 1.59 | 1.00 |
| 1991 |  | 394 | 233 | 161 | 13904391 | 1.68 | 1.16 |
| 1992 |  | 403 | 247 | 156 | 14055134 | 1.76 | 1.11 |
| 1993 |  | 358 | 227 | 131 | 14205297 | 1.60 | 0.92 |
| 1994 |  | 327 | 191 | 136 | 14357184 | 1.33 | 0.95 |
| 1995 |  | 330 | 216 | 114 | 14502481 | 1.49 | 0.79 |
| 1996 |  | 344 | 219 | 125 | 14650314 | 1.49 | 0.85 |
| 1997 |  | 285 | 170 | 115 | 14806454 | 1.15 | 0.78 |
| 1998 |  | 312 | 211 | 101 | 14925127 | 1.41 | 0.68 |
| 1999 |  | 319 | 207 | 112 | 15048669 | 1.38 | 0.74 |
| 2000 |  | 339 | 213 | 126 | 15193812 | 1.40 | 0.83 |
| 2001 |  | 336 | 217 | 119 | 15366600 | 1.41 | 0.77 |
| 2002 |  | 316 | 206 | 110 | 15537576 | 1.33 | 0.71 |
| 2003 |  | 326 | 214 | 112 | 15679310 | 1.36 | 0.71 |
| 2004 |  | 367 | 242 | 125 | 15827090 | 1.53 | 0.79 |
| 2005 |  | 429 | 259 | 170 | 15980008 | 1.62 | 1.06 |
| 2006 |  | 362 | 229 | 133 | 16144759 | 1.42 | 0.82 |
| 2007 |  | 382 | 241 | 141 | 16298852 | 1.48 | 0.87 |
| 2008 |  | 452 | 277 | 175 | 16474178 | 1.68 | 1.06 |
| 2009 |  | 430 | 281 | 149 | 16663413 | 1.69 | 0.89 |
| 2010 |  | 389 | 246 | 143 | 16847823 | 1.46 | 0.85 |
| 2011 |  | 380 | 263 | 117 | 17014528 | 1.55 | 0.69 |
| 2012 |  | 361 | 226 | 135 | 17209900 | 1.31 | 0.78 |
| 2013 |  | 318 | 222 | 96 | 17401165 | 1.28 | 0.55 |
| 2014 |  | 324 | 220 | 104 | 17581697 | 1.25 | 0.59 |
| 2015 |  | 322 | 215 | 107 | 17712801 | 1.21 | 0.60 |
| 2016 |  | 303 | 182 | 121 | 17916496 | 1.02 | 0.68 |
|  |  |  |  |  |  |  |  |
| **Female Homicide** | | |  |  |  |  |  |
| 1981 |  | 214 | 157 | 57 | 12468682 | 1.26 | 0.46 |
| 1982 |  | 194 | 130 | 64 | 12626241 | 1.03 | 0.51 |
| 1983 |  | 207 | 147 | 60 | 12759682 | 1.15 | 0.47 |
| 1984 |  | 184 | 131 | 53 | 12889230 | 1.02 | 0.41 |
| 1985 |  | 197 | 143 | 54 | 13014010 | 1.10 | 0.41 |
| 1986 |  | 175 | 120 | 55 | 13148901 | 0.91 | 0.42 |
| 1987 |  | 196 | 128 | 68 | 13321475 | 0.96 | 0.51 |
| 1988 |  | 166 | 122 | 44 | 13502708 | 0.90 | 0.33 |
| 1989 |  | 202 | 136 | 66 | 13752280 | 0.99 | 0.48 |
| 1990 |  | 199 | 150 | 49 | 13969405 | 1.07 | 0.35 |
| 1991 |  | 228 | 148 | 80 | 14133029 | 1.05 | 0.57 |
| 1992 |  | 194 | 136 | 58 | 14316130 | 0.95 | 0.41 |
| 1993 |  | 168 | 124 | 44 | 14479467 | 0.86 | 0.30 |
| 1994 |  | 171 | 135 | 36 | 14643479 | 0.92 | 0.25 |
| 1995 |  | 159 | 121 | 38 | 14799830 | 0.82 | 0.26 |
| 1996 |  | 167 | 113 | 54 | 14959904 | 0.76 | 0.36 |
| 1997 |  | 146 | 102 | 44 | 15099494 | 0.68 | 0.29 |
| 1998 |  | 155 | 126 | 29 | 15230046 | 0.83 | 0.19 |
| 1999 |  | 143 | 104 | 39 | 15352617 | 0.68 | 0.25 |
| 2000 |  | 128 | 98 | 30 | 15491918 | 0.63 | 0.19 |
| 2001 |  | 127 | 98 | 29 | 15654302 | 0.63 | 0.19 |
| 2002 |  | 146 | 119 | 27 | 15822503 | 0.75 | 0.17 |
| 2003 |  | 121 | 95 | 26 | 15964718 | 0.60 | 0.16 |
| 2004 |  | 148 | 124 | 24 | 16113565 | 0.77 | 0.15 |
| 2005 |  | 147 | 115 | 32 | 16263745 | 0.71 | 0.20 |
| 2006 |  | 125 | 92 | 33 | 16426415 | 0.56 | 0.20 |
| 2007 |  | 133 | 107 | 26 | 16590173 | 0.64 | 0.16 |
| 2008 |  | 123 | 101 | 22 | 16772940 | 0.60 | 0.13 |
| 2009 |  | 144 | 116 | 28 | 16965482 | 0.68 | 0.17 |
| 2010 |  | 135 | 106 | 29 | 17157066 | 0.62 | 0.17 |
| 2011 |  | 165 | 135 | 30 | 17324800 | 0.78 | 0.17 |
| 2012 |  | 132 | 106 | 26 | 17504322 | 0.61 | 0.15 |
| 2013 |  | 128 | 106 | 22 | 17681789 | 0.60 | 0.12 |
| 2014 |  | 115 | 84 | 31 | 17855738 | 0.47 | 0.17 |
| 2015 |  | 134 | 104 | 30 | 17990107 | 0.58 | 0.17 |
| 2016 |  | 93 | 76 | 17 | 18192991 | 0.42 | 0.09 |
