# Supplementary material for: Effect of firearms legislation on suicide and homicide in Canada from 1981 to 2016
Source: PLoS One. 2020 Jun 18;15(6):e0234457. doi: 10.1371/journal.pone.0234457 (PMC7302582; doi:10.1371/journal.pone.0234457)
Supplement: S4 Table — Results of regression model are expressed as regression coefficients, percentage change per year of suicide rates. (DOCX) [file pone.0234457.s004.docx]

| **Variable** | **β coefficient**  **(95% CI)** | **P** |  | **Percent Change**  **(95% CI)** |
| --- | --- | --- | --- | --- |
|  |  |  |  |  |
| **Aggregate Male Suicide** |  |  |  |  |
|  |  |  |  |  |
| **1991 Safe Storage** |  |  |  |  |
| Trend in Hanging mortality before law | 0.007 (-0.005, 0.020) | 0.24 | Hanging mortality before law^1^ | 0.738% (-0.494%, -1.994%) |
| Additional trend in firearm mortality before law | -0.030 (-0.046, -0.014) | <0.01 | Firearm mortality before law^1^ | -2.277% (-3.238%, -1.325%) |
| Change in trend in hanging mortality after law | -0.004 (-0.017, 0.009) | 0.56 | Hanging mortality after law^1^ | 0.360% (-0.001%, 0.719%) |
| Additional change in trend of firearm mortality after law | -0.013 (-0.030, 0.005) | 0.16 | Firearm mortality after law^1^ | -3.981% (-4.753%, -3.215%) |
| Rate ratio of mortality at start | 0.508 (0.393, 0.627) | <0.01 | Rate ratio of mortality at start | 39.856% (32.506%, 46.601%) |
| Hanging level effect | 0.264 (0.132, 0.397) | <0.01 | Hanging level effect | 23.221% (12.331%, 32.759%) |
| Firearms level effect | 0.087 (-0.405, 0.051) | 0.37 | Firearms level effect | 8.342% (-49.905%, 4.93%) |
|  |  |  |  |  |
| **1994 Psychiatric Questionnaire** |  |  |  |  |
| Trend in Hanging mortality before law | 0.016 (0.004, 0.028) | 0.01 | Hanging mortality before law^1^ | 1.571% (0.403%, 2.725%) |
| Additional trend in firearm mortality before law | -0.038 (-0.051, -0.024) | <0.01 | Firearm mortality before law^1^ | -2.214% (-2.878%, -1.554%) |
| Change in trend in hanging mortality after law | -0.014 (-0.027, -0.002) | 0.03 | Hanging mortality after law^1^ | 0.165% (-0.222%, 0.552%) |
| Additional change in trend of firearm mortality after law | 0.000 (-0.016, 0.016) | 0.98 | Firearm mortality after law^1^ | -1.240% (-2.364%, -0.130%) |
| Rate ratio of mortality at start | 0.534 (0.433, 0.654) | <0.01 | Rate ratio of mortality at start | 41.382% (35.112%, 47.991%) |
| Hanging level effect | 0.357 (0.214, 0.501) | <0.01 | Hanging level effect | 30.044% (19.25%, 39.394%) |
| Firearms level effect | -0.001 (-0.207, 0.204) | 0.99 | Firearms level effect | -0.141% (-23.032%, 18.491%) |
|  |  |  |  |  |
| **2001 Licensing** |  |  |  |  |
|  |  |  |  |  |
| Trend in Hanging mortality before law | 0.025 (0.018, 0.031) | <0.01 | Hanging mortality before law^1^ | 2.453% (1.802%, 3.099%) |
| Additional trend in firearm mortality before law | -0.060 (-0.069, -0.051) | <0.01 | Firearm mortality before law^1^ | -3.597% (-4.275%, -2.923%) |
| Change in trend in hanging mortality after law | -0.022 (-0.031, -0.013) | <0.01 | Hanging mortality after law^1^ | 0.284% (-0.267%, 0.831%) |
| Additional change in trend of firearm mortality after law | 0.040 (0.026, 0.053) | <0.01 | Firearm mortality after law^1^ | -1.787% (-2.672%, -0.909%) |
| Rate ratio of mortality at start | 0.674 (0.569, 0.779) | <0.01 | Rate ratio of mortality at start | 49.029% (43.381%, 54.114%) |
| Hanging level effect | 0.367 (0.193, 0.542) | <0.01 | Hanging level effect | 30.733% (17.53%, 41.822%) |
| Firearms level effect | -0.635 (-0.888, -0.381) | <0.01 | Firearms level effect | -88.66% (-143.143%, -46.386%) |
|  |  |  |  |  |
| **Male Age 15 to 29** |  |  |  |  |
|  |  |  |  |  |
| **1991 Safe Storage** |  |  |  |  |
| Trend in Hanging mortality before law | 0.034 (0.011, 0.057) | <0.01 | Hanging mortality before law^1^ | 3.345% (1.118%, 5.522%) |
| Additional trend in firearm mortality before law | -0.061 (-0.088, -0.034) | <0.01 | Firearm mortality before law^1^ | -2.709% (-4.158%, -1.235%) |
| Change in trend in hanging mortality after law | -0.041 (-0.064, -0.018) | <0.01 | Hanging mortality after law^1^ | -0.716% (-1.201%, -0.233%) |
| Additional change in trend of firearm mortality after law | 0.005 (-0.026, 0.037) | 0.74 | Firearm mortality after law^1^ | -6.450% (-8.104%, -4.822%) |
| Rate ratio of mortality at start | 0.661 (0.461, 0.86) | <0.01 | Rate ratio of mortality at start | 48.343% (36.955%, 57.672%) |
| Hanging level effect | 0.654 (0.447, 0.861) | <0.01 | Hanging level effect | 48.005% (36.021%, 57.744%) |
| Firearms level effect | 0.219 (-0.158, 0.597) | 0.25 | Firearms level effect | 19.705% (-17.075%, 44.931%) |
|  |  |  |  |  |
| **1994 Psychiatric Questionnaire** |  |  |  |  |
| Trend in Hanging mortality before law | 0.035 (0.021, 0.050) | <0.01 | Hanging mortality before law^1^ | 3.454% (2.035%, 4.853%) |
| Additional trend in firearm mortality before law | -0.058 (-0.077, -0.039) | <0.01 | Firearm mortality before law^1^ | -2.288% (-3.596%, -0.997%) |
| Change in trend in hanging mortality after law | -0.045 (-0.060, -0.030) | <0.01 | Hanging mortality after law^1^ | -0.998% (-1.470%, -0.528%) |
| Additional change in trend of firearm mortality after law | 0.013 (-0.012, 0.038) | 0.30 | Firearm mortality after law^1^ | -5.617% (-7.232%, -4.027%) |
| Rate ratio of mortality at start | 0.649 (0.479, 0.818) | <0.01 | Rate ratio of mortality at start | 47.724% (38.082%, 55.864%) |
| Hanging level effect | 0.739 (0.557, 0.921) | <0.01 | Hanging level effect | 52.225% (42.691%, 60.173%) |
| Firearms level effect | 0.008 (-0.369, 0.384) | 0.97 | Firearms level effect | 0.752% (-44.625%, 31.891%) |
|  |  |  |  |  |
| **2001 Licensing** |  |  |  |  |
|  |  |  |  |  |
| Trend in Hanging mortality before law | 0.033 (0.026, 0.041) | <0.01 | Hanging mortality before law^1^ | 3.270% (2.543%, 3.993%) |
| Additional trend in firearm mortality before law | -0.088 (-0.103, -0.073) | <0.01 | Firearm mortality before law^1^ | -5.591% (-6.965%, -4.234%) |
| Change in trend in hanging mortality after law | -0.042 (-0.053, -0.031) | <0.01 | Hanging mortality after law^1^ | -0.858% (-1.671%, -0.053%) |
| Additional change in trend of firearm mortality after law | 0.073 (0.047, 0.099) | <0.01 | Firearm mortality after law^1^ | -2.311% (-4.405%, -0.259%) |
| Rate ratio of mortality at start | 0.825 (0.658, 0.993) | <0.01 | Rate ratio of mortality at start | 56.19% (48.196%, 62.951%) |
| Hanging level effect | 0.678 (0.427, 0.929) | <0.01 | Hanging level effect | 49.247% (34.762%, 60.515%) |
| Firearms level effect | -1.156 (-1.74, -0.573) | <0.01 | Firearms level effect | -217.8% (-469.686%, -77.285%) |
|  |  |  |  |  |
| **Male Age 30 to 44** |  |  |  |  |
|  |  |  |  |  |
| **1991 Safe Storage** |  |  |  |  |
| Trend in Hanging mortality before law | 0.024 (.008, 0.040) | <0.01 | Hanging mortality before law^1^ | 2.385% (0.824%, 3.921%) |
| Additional trend in firearm mortality before law | -0.039 (-0.058, -0.020) | <0.01 | Firearm mortality before law^1^ | -1.483% (-2.504%, -0.472%) |
| Change in trend in hanging mortality after law | -0.021 (-0.037, -0.004) | 0.02 | Hanging mortality after law^1^ | 0.360% (-0.141%, 0.858%) |
| Additional change in trend of firearm mortality after law | -0.016 (-0.036, 0.004) | 0.12 | Firearm mortality after law^1^ | -5.249% (-6.02%, -4.484%) |
| Rate ratio of mortality at start | 0.521 (0.388, 0.655) | <0.01 | Rate ratio of mortality at start | 40.613% (32.128%, 48.038%) |
| Hanging level effect | 0.574 (0.395, 0.752) | <0.01 | Hanging level effect | 43.648% (32.654%, 52.847%) |
| Firearms level effect | 0.343 (0.158, 0.528) | <0.01 | Firearms level effect | 29.008% (14.586%, 40.995%) |
|  |  |  |  |  |
| **1994 Psychiatric Questionnaire** |  |  |  |  |
| Trend in Hanging mortality before law | 0.037 (0.021, 0.052) | <0.01 | Hanging mortality before law^1^ | 3.593% (2.083%, 5.08%) |
| Additional trend in firearm mortality before law | -0.053 (-0.070, -0.036) | <0.01 | Firearm mortality before law^1^ | -1.615% (-2.289%, -0.946%) |
| Change in trend in hanging mortality after law | -0.036 (-0.052, -0.019) | <0.01 | Hanging mortality after law^1^ | 0.072% (-0.450%, 0.591%) |
| Additional change in trend of firearm mortality after law | 0.003 (-0.017, 0.022) | 0.74 | Firearm mortality after law^1^ | -5.024% (-5.917%, -4.139%) |
| Rate ratio of mortality at start | 0.582 (0.448, 0.716) | <0.01 | Rate ratio of mortality at start | 44.136% (36.122%, 51.145%) |
| Hanging level effect | 0.711 (0.517, 0.905) | <0.01 | Hanging level effect | 50.88% (40.349%, 59.553%) |
| Firearms level effect | 0.275 (0.055, 0.496) | 0.01 | Firearms level effect | 24.062% (5.331%, 39.087%) |
|  |  |  |  |  |
| **2001 Licensing** |  |  |  |  |
|  |  |  |  |  |
| Trend in Hanging mortality before law | 0.043 (0.036, 0.050) | <0.01 | Hanging mortality before law^1^ | 4.210% (3.495%, 4.920%) |
| Additional trend in firearm mortality before law | -0.076 (-0.087, -0.065) | <0.01 | Firearm mortality before law^1^ | -3.327% (-4.201%, -2.460%) |
| Change in trend in hanging mortality after law | -0.046 (-0.056, -0.036) | <0.01 | Hanging mortality after law^1^ | -0.294% (-0.910%, 0.319%) |
| Additional change in trend of firearm mortality after law | 0.049 (0.031, 0.067) | <0.01 | Firearm mortality after law^1^ | -3.044% (-4.33%, -1.775%) |
| Rate ratio of mortality at start | 0.718 (0.592, 0.845) | <0.01 | Rate ratio of mortality at start | 51.252% (44.654%, 57.063%) |
| Hanging level effect | 0.850 (0.643, 1.058) | <0.01 | Hanging level effect | 57.261% (47.404%, 65.27%) |
| Firearms level effect | -0.406 (-0.785, -0.027) | 0.04 | Firearms level effect | -50.124% (-119.337%, -2.752%) |
|  |  |  |  |  |
| **Male Age 45 to 59** |  |  |  |  |
|  |  |  |  |  |
| **1991 Safe Storage** |  |  |  |  |
| Trend in Hanging mortality before law | -0.028 (-0.054, -0.001) | 0.04 | Hanging mortality before law^1^ | -2.79% (-5.522%, -0.128%) |
| Additional trend in firearm mortality before law | -0.002 (-0.032, 0.029) | 0.92 | Firearm mortality before law^1^ | -2.951% (-4.509%, -1.416%) |
| Change in trend in hanging mortality after law | 0.051 (0.025, 0.077) | <0.01 | Hanging mortality after law^1^ | 2.32% (1.914%, 2.724%) |
| Additional change in trend of firearm mortality after law | -0.057 (-0.088, -0.026) | <0.01 | Firearm mortality after law^1^ | -3.565% (-4.091%, -3.042%) |
| Rate ratio of mortality at start | 0.482 (0.295, 0.669) | <0.01 | Rate ratio of mortality at start | 38.246% (25.533%, 48.788%) |
| Hanging level effect | -0.290 (-0.484, -0.096) | <0.01 | Hanging level effect | -33.632% (-62.255%, -10.058%) |
| Firearms level effect | 0.069 (-0.074, 0.212) | 0.34 | Firearms level effect | 6.655% (-7.666%, 19.07%) |
|  |  |  |  |  |
| **1994 Psychiatric Questionnaire** |  |  |  |  |
| Trend in Hanging mortality before law | -0.007 (-0.033, 0.018) | 0.58 | Hanging mortality before law^1^ | -0.735% (-3.345%, 1.809%) |
| Additional trend in firearm mortality before law | -0.020 (-0.048, 0.008) | 0.17 | Firearm mortality before law^1^ | -2.731% (-3.914%, -1.560%) |
| Change in trend in hanging mortality after law | 0.030 (0.004, 0.057) | 0.02 | Hanging mortality after law^1^ | 2.265% (1.742%, 2.785%) |
| Additional change in trend of firearm mortality after law | -0.038 (-0.067, -0.009) | 0.01 | Firearm mortality after law^1^ | -3.489% (-4.054%, -2.927%) |
| Rate ratio of mortality at start | 0.559 (0.356, 0.761) | <0.01 | Rate ratio of mortality at start | 42.809% (29.984%, 53.286%) |
| Hanging level effect | -0.186 (-0.43, 0.058) | 0.14 | Hanging level effect | -20.483% (-53.785%, 5.608%) |
| Firearms level effect | 0.058 (-0.085, 0.201) | 0.43 | Firearms level effect | 5.652% (-8.847%, 18.219%) |
|  |  |  |  |  |
| **2001 Licensing** |  |  |  |  |
|  |  |  |  |  |
| Trend in Hanging mortality before law | 0.017 (0.003, 0.031) | 0.02 | Hanging mortality before law^1^ | 1.670% (0.250%, 3.070%) |
| Additional trend in firearm mortality before law | -0.047 (-0.062, -0.032) | <0.01 | Firearm mortality before law^1^ | -3.081% (-3.563%, -2.601%) |
| Change in trend in hanging mortality after law | 0.005 (-0.012, 0.019) | 0.59 | Hanging mortality after law^1^ | 2.120% (1.318%, 2.916%) |
| Additional change in trend of firearm mortality after law | 0.000 (-0.019, 0.019) | 0.97 | Firearm mortality after law^1^ | -2.644% (-3.544%, -1.750%) |
| Rate ratio of mortality at start | 0.717 (0.528, 0.906) | <0.01 | Rate ratio of mortality at start | 51.169% (40.997%, 59.587%) |
| Hanging level effect | -0.009 (-0.304, 0.286) | 0.95 | Hanging level effect | -0.944% (-35.587%, 24.848%) |
| Firearms level effect | -0.213 (-0.459, 0.034) | 0.09 | Firearms level effect | -23.711% (-58.326%, 3.337%) |
|  |  |  |  |  |
| **Male Age 60 plus** |  |  |  |  |
|  |  |  |  |  |
| **1991 Safe Storage** |  |  |  |  |
| Trend in Hanging mortality before law | -0.040 (-0.067, -0.013) | <0.01 | Hanging mortality before law^1^ | -4.066% (-6.939%, -1.270%) |
| Additional trend in firearm mortality before law | 0.025 (-0.007, 0.058) | 0.13 | Firearm mortality before law^1^ | -1.448% (-3.300%, 0.371%) |
| Change in trend in hanging mortality after law | 0.044 (0.017, 0.072) | <0.01 | Hanging mortality after law^1^ | 0.463% (0.0370%, 0.887%) |
| Additional change in trend of firearm mortality after law | -0.055 (-0.088, -0.022) | <0.01 | Firearm mortality after law^1^ | -2.525% (-3.101%, -1.952%) |
| Rate ratio of mortality at start | 0.204 (-0.037, 0.445) | 0.10 | Rate ratio of mortality at start | 18.457% (-3.725%, 35.895%) |
| Hanging level effect | -0.514 (-0.752, -0.277) | <0.01 | Hanging level effect | -67.227% (-112.073%, -31.864%) |
| Firearms level effect | 0.048 (-0.128, 0.225) | 0.59 | Firearms level effect | 4.73% (-13.674%, 20.155%) |
|  |  |  |  |  |
| **1994 Psychiatric Questionnaire** |  |  |  |  |
| Trend in Hanging mortality before law | -0.037 (-0.056, -0.018) | <0.01 | Hanging mortality before law^1^ | -3.743% (-5.724%, -1.798%) |
| Additional trend in firearm mortality before law | 0.018 (-0.004, 0.041) | 0.11 | Firearm mortality before law^1^ | -1.854% (-3.194%, -0.530%) |
| Change in trend in hanging mortality after law | 0.042 (0.022, 0.061) | <0.01 | Hanging mortality after law^1^ | 0.479% (-0.090%, 1.044%) |
| Additional change in trend of firearm mortality after law | -0.048 (-0.072, -0.024) | <0.01 | Firearm mortality after law^1^ | -2.500% (-3.186%, -1.819%) |
| Rate ratio of mortality at start | 0.235 (0.034, 0.437) | 0.02 | Rate ratio of mortality at start | 20.977% (3.34%, 35.396%) |
| Hanging level effect | -0.505 (-0.741, -0.268) | <0.01 | Hanging level effect | -65.619% (-109.798%, -30.743%) |
| Firearms level effect | 0.024 (-0.166, 0.215) | 0.80 | Firearms level effect | 2.395% (-18.073%, 19.315%) |
|  |  |  |  |  |
| **2001 Licensing** |  |  |  |  |
|  |  |  |  |  |
| Trend in Hanging mortality before law | -0.018 (-0.029, -0.007) | <0.01 | Hanging mortality before law^1^ | -1.812% (-2.974%, -0.663%) |
| Additional trend in firearm mortality before law | -0.003 (-0.016, 0.009) | 0.59 | Firearm mortality before law^1^ | -2.161% (-2.715%, -1.609%) |
| Change in trend in hanging mortality after law | 0.033 (0.018, 0.048) | <0.01 | Hanging mortality after law^1^ | 1.505% (0.618%, 2.383%) |
| Additional change in trend of firearm mortality after law | -0.023 (-0.042, -0.004) | 0.02 | Firearm mortality after law^1^ | -1.134% (-2.203%, -0.075%) |
| Rate ratio of mortality at start | 0.365 (0.208, 0.521) | <0.01 | Rate ratio of mortality at start | 30.567% (18.819%, 40.615%) |
| Hanging level effect | -0.711 (-1.102, -0.405) | <0.01 | Hanging level effect | -103.696% (-200.946%, -49.978%) |
| Firearms level effect | -0.436 (-0.712, -0.095) | 0.01 | Firearms level effect | -54.574% (-103.884%, -9.935%) |
|  |  |  |  |  |
| **Aggregate Female Suicide** |  |  |  |  |
|  |  |  |  |  |
| **1991 Safe Storage** |  |  |  |  |
| Trend in Hanging mortality before law | -0.018 (-0.045, 0.008) | 0.17 | Hanging mortality before law^1^ | -1.862% (-4.554%, 0.760%) |
| Additional trend in firearm mortality before law | -0.023 (-0.058, 0.011) | 0.19 | Firearm mortality before law^1^ | -4.271% (-6.675%, -1.920%) |
| Change in trend in hanging mortality after law | 0.037 (0.011, 0.063) | 0.01 | Hanging mortality after law^1^ | 1.836% (1.472%, 2.199%) |
| Additional change in trend of firearm mortality after law | -0.055 (-0.093, -0.016) | 0.01 | Firearm mortality after law^1^ | -6.121% (-7.927%, -4.345%) |
| Rate ratio of mortality at start | -0.572 (-0.803, -0.341) | <0.01 | Rate ratio of mortality at start | -77.18% (-123.123%, -40.697%) |
| Hanging level effect | -0.104 (-0.294, 0.086) | 0.28 | Hanging level effect | -10.945% (-34.183%, 8.268%) |
| Firearms level effect | 0.008 (-0.372, 0.388) | 0.97 | Firearms level effect | 0.797% (-45.111%, 32.181%) |
|  |  |  |  |  |
| **1994 Psychiatric Questionnaire** |  |  |  |  |
| Trend in Hanging mortality before law | 0.000 (-0.024, 0.024) | 0.98 | Hanging mortality before law^1^ | 0.036% (-2.379%, 2.394%) |
| Additional trend in firearm mortality before law | -0.048 (-0.076, -0.019) | <0.01 | Firearm mortality before law^1^ | -4.846% (-6.448%, -3.269%) |
| Change in trend in hanging mortality after law | 0.017 (-0.008, 0.041) | 0.18 | Hanging mortality after law^1^ | 1.675% (1.286%, 2.063%) |
| Additional change in trend of firearm mortality after law | -0.026 (-0.060, 0.008) | 0.14 | Firearm mortality after law^1^ | -5.841% (-7.939%, -3.785%) |
| Rate ratio of mortality at start | -0.464 (-0.671, -0.257) | <0.01 | Rate ratio of mortality at start | -59.049% (-95.575%, -29.344%) |
| Hanging level effect | 0.027 (-0.156, 0.209) | 0.78 | Hanging level effect | 2.616% (-16.882%, 18.862%) |
| Firearms level effect | -0.093 (-0.554, 0.368) | 0.69 | Firearms level effect | -9.763% (-74.031%, 30.771%) |
|  |  |  |  |  |
| **2001 Licensing** |  |  |  |  |
|  |  |  |  |  |
| Trend in Hanging mortality before law | 0.025 (0.014, 0.036) | <0.01 | Hanging mortality before law^1^ | 2.500% (1.434%, 3.554%) |
| Additional trend in firearm mortality before law | -0.083 (-0.098, -0.069) | <0.01 | Firearm mortality before law^1^ | -5.962% (-7.056%, -4.880%) |
| Change in trend in hanging mortality after law | -0.001 (-0.014, 0.011) | 0.84 | Hanging mortality after law^1^ | 2.375% (1.750%, 2.996%) |
| Additional change in trend of firearm mortality after law | 0.035 (-0.004, 0.074) | 0.08 | Firearm mortality after law^1^ | -2.423% (-6.099%, 1.127%) |
| Rate ratio of mortality at start | -0.26 (-0.454, -0.067) | 0.01 | Rate ratio of mortality at start | -29.715% (-57.394%, -6.903%) |
| Hanging level effect | -0.057 (-0.296, 0.182) | 0.64 | Hanging level effect | -5.866% (-34.406%, 16.614%) |
| Firearms level effect | -1.111 (-2.158, -0.063) | 0.04 | Firearms level effect | -203.604% (-765.586%, -6.489%) |
|  |  |  |  |  |

^1^Percent change per year
