# Supplementary material for: Effect of firearms legislation on suicide and homicide in Canada from 1981 to 2016
Source: PLoS One. 2020 Jun 18;15(6):e0234457. doi: 10.1371/journal.pone.0234457 (PMC7302582; doi:10.1371/journal.pone.0234457)
Supplement: S5 Table — Results of linear combination calculations of post effect rate ratios for non-firearm and firearms suicide demonstrating method substitution of firearm suicide with hanging. Linear combinations of the addition of the regression coefficients, if statistically equal, should result in a rate ratio of ~1.0 with confidence intervals crossing 1.0. (DOCX) [file pone.0234457.s005.docx]

| **Variable** | **Combination of post effect trend, rate ratio**  **(95% CI)** | **P** |
| --- | --- | --- |
|  |  |  |
| **Suicide** |  |  |
|  |  |  |
| Male Age 45 to 59, 1991 | 0.994 (0.978, 1.009) | 0.46 |
| Male Age 45 to 59, 1994 | 0.993 (0.980, 1.005) | 0.25 |
| Male Age 60+, 1991 | 0.989 (0.971, 1.008) | 0.28 |
| Male Age 60+, 1994 | 0.994 (0.979, 1.008) | 0.40 |
| Male Age 60+, 2001 | 1.010 (0.998, 1.022) | 0.09 |
| Female, 1991 | 0.984 (0.957, 1.011) | 0.26 |

Results of linear combination calculations of post effect rate ratios for all non firearm suicide and firearm suicide demonstrating method substitution of firearm suicide with non firearm suicide. Linear combinations of the addition of the regression coefficients, if statistically equal, should result in a rate ratio of ~1.0 with confidence intervals crossing 1.0.
