# Supplementary material for: Effect of firearms legislation on suicide and homicide in Canada from 1981 to 2016
Source: PLoS One. 2020 Jun 18;15(6):e0234457. doi: 10.1371/journal.pone.0234457 (PMC7302582; doi:10.1371/journal.pone.0234457)
Supplement: S6 Table — Results of sensitivity tests performed using non hanging non firearm data for males and suicide by jumping data for females. No associated decreased in the rate of suicide by firearm was found in males 45 years and older and all females after 1991 suggesting that suicide by hanging had replaced firearms as a method in the cohorts and interventions where an effect was found in the DiD model. 1The rate ratio of the trend of firearm mortality after each year of legislation implementation which is the difference-in-difference regression result. A rate ratio greater than 1 suggests that firearm mortality by suicide or homicide is increasing greater than mortality by other methods, while a ratio less than 1 suggests there is a decrease greater than other methods. 2Additional change in trend. (DOCX) [file pone.0234457.s006.docx]

| **Age and Gender Cohort by Effect Year** | **Suicide Rate Ratio^1^**  **(95% CI)** |
| --- | --- |
|  |  |
| **Male Age 45 to 59** |  |
|  |  |
| **1991 Safe Storage** |  |
| firearm mortality after law^2^ | 0.979 (0.953, 1.006) |
|  |  |
| **1994 Psychiatric Questionnaire** |  |
| firearm mortality after law^2^ | 0.986 (0.966, 1.006) |
|  |  |
| **Male Age 60 plus** |  |
|  |  |
| **1991 Safe Storage** | 0.983 (0.950, 1.018) |
| firearm mortality after law^2^ |  |
|  |  |
| **1994 Psychiatric Questionnaire** |  |
| firearm mortality after law^2^ | 0.984 (0.961, 1.007) |
|  |  |
| **2001 Licensing** |  |
| firearm mortality after law^2^ | 0.984 (0.967, 1.002) |
|  |  |
| **Aggregate Female** |  |
|  |  |
| **1991 Safe Storage** |  |
| firearm mortality after law^2^ | 0.970 (0.924, 1.019) |
|  |  |

^1^The rate ratio of the trend of firearm mortality after each year of legislation implementation which is the difference-in-difference regression result. A rate ratio greater than 1 suggests that firearm mortality by suicide or homicide is increasing greater than mortality by other methods, while a ratio less than 1 suggests there is a decrease greater than other methods.

^2^Additional change in trend
