# Supplementary material for: Effect of firearms legislation on suicide and homicide in Canada from 1981 to 2016
Source: PLoS One. 2020 Jun 18;15(6):e0234457. doi: 10.1371/journal.pone.0234457 (PMC7302582; doi:10.1371/journal.pone.0234457)
Supplement: S7 Table — Results of regression model are expressed as regression coefficients, percentage change per year of homicide rates. (DOCX) [file pone.0234457.s007.docx]

| **Variable** | **β coefficient**  **(95% CI)** | **P** |  | **Percent Change**  **(95% CI)** |
| --- | --- | --- | --- | --- |
|  |  |  |  |  |
| **Male Homicide** |  |  |  |  |
|  |  |  |  |  |
| **1991 Safe Storage** |  |  |  |  |
| Trend in non firearm homicide before law | 0.075 (-0.098, 0.247) | 0.40 | Non firearm homicide before law^1^ | 7.191% (-10.308%, 21.914%) |
| Additional trend in firearm homicide before law | 0.006 (-0.030, 0.043) | 0.74 | Firearm homicide before law^1^ | 0.622% (-3.073%, 4.185%) |
| Change in trend in non firearm homicide after law | -0.137 (-0.230 -0.045) | <0.01 | Non firearm homicide after law^1^ | -6.483% (-23.583%, 8.251%) |
| Additional change in trend of firearm homicide after law | -0.012 (-0.049, 0.026) | 0.55 | Firearm homicide after law^1^ | -7.049% (-24.192%, 7.728%) |
| Rate ratio of mortality at start | -0.564 (-0.798, -0.33) | <0.01 | Rate ratio of mortality at start | -75.731% (-122.106%, -39.04%) |
| Non firearm level effect | 1.583 (0.655, 2.511) | <0.01 | Non firearm level effect | 79.465% (48.036%, 91.885%) |
| Firearm level effect | 1.698 (0.719, 2.677) | <0.01 | Firearm level effect | 81.691% (51.263%, 93.122%) |
|  |  |  |  |  |
| **1994 Psychiatric Questionnaire** |  |  |  |  |
| Trend in non firearm homicide before law | 0.023 (-0.143, 0.188) | 0.79 | Non firearm homicide before law^1^ | 2.227% (-15.387%, 17.152%) |
| Additional trend in firearm homicide before law | 0.005 (-0.021, 0.031) | 0.70 | Firearm homicide before law^1^ | 2.731% (-14.406%, 17.302%) |
| Change in trend in non firearm homicide after law | -0.085 (-0.158, -0.013) | 0.02 | Non firearm homicide after law^1^ | -6.484% (-21.712%, 6.838%) |
| Additional change in trend of firearm homicide after law | -0.010 (-0.039, 0.018) | 0.49 | Firearm homicide after law^1^ | -7.015% (-22.214%, 6.294%) |
| Rate ratio of mortality at start | -0.559 (-0.753, -0.365) | <0.01 | Rate ratio of mortality at start | -74.849% (-112.304%, -44.002%) |
| Non firearm level effect | 1.010 (0.05, 1.97) | 0.04 | Non firearm level effect | 63.579% (4.915%, 86.049%) |
| Firearm level effect | 1.111 (0.163, 2.059) | 0.02 | Firearm level effect | 67.07% (15.025%, 87.239%) |
|  |  |  |  |  |
| **2001 Licensing** |  |  |  |  |
|  |  |  |  |  |
| Trend in non firearm homicide before law | -0.085 (-0.252, 0.082) | 0.32 | Non firearm homicide before law^1^ | -8.886% (-28.704%, 7.88%) |
| Additional trend in firearm homicide before law | -0.001 (-0.012, 0.010) | 0.86 | Firearm homicide before law^1^ | -8.998% (-28.744%, 7.719%) |
| Change in trend in non firearm homicide after law | 0.079 (-0.036, 0.194) | 0.18 | Non firearm homicide after law^1^ | -0.606% (-28.732%, 21.375%) |
| Additional change in trend of firearm homicide after law | -0.003 (-0.027, 0.021) | 0.82 | Firearm homicide after law^1^ | -0.989% (-28.952%, 20.911%) |
| Rate ratio of mortality at start | -0.52 (-0.643, -0.396) | <0.01 | Rate ratio of mortality at start | -68.141% (-90.28%, -48.577%) |
| Non firearm level effect | -1.618 (-3.936, 0.7) | 0.17 | Non firearm level effect | -404.382% (-5023.844%, 50.35%) |
| Firearm level effect | -1.589 (-3.844, 0.665) | 0.17 | Firearm level effect | -389.929% (-4569.644%, 48.598%) |
|  |  |  |  |  |
| **Female Homicide** |  |  |  |  |
|  |  |  |  |  |
| **1991 Safe Storage** |  |  |  |  |
| Trend in non firearm homicide before law | -0.144 (-0.398, 0.111) | 0.27 | Non firearm homicide before law^1^ | -15.453% (-48.905%, 10.483%) |
| Additional trend in firearm homicide before law | 0.0120 (-0.029, 0.053) | 0.58 | Firearm homicide before law^1^ | -14.107% (-46.947%, 11.394%) |
| Change in trend in non firearm homicide after law | -0.084 (-0.224, 0.056) | 0.24 | Non firearm homicide after law^1^ | -25.556% (-52.325%, -3.492%) |
| Additional change in trend of firearm homicide after law | -0.029 (-0.072, 0.014) | 0.18 | Firearm homicide after law^1^ | -27.798% (-55.337%, -5.14%) |
| Rate ratio of mortality at start | -0.915 (-1.129, -0.701) | <0.01 | Rate ratio of mortality at start | -149.656% (-209.355%, -101.478%) |
| Non firearm level effect | 0.919 (-0.479, 2.317) | 0.20 | Non firearm level effect | 60.125% (-61.386%, 90.148%) |
| Firearm level effect | 1.012 (-0.419, 2.443) | 0.17 | Firearm level effect | 63.663% (-51.969%, 91.312%) |
|  |  |  |  |  |
| **1994 Psychiatric Questionnaire** |  |  |  |  |
| Trend in non firearm homicide before law | -0.207 (-0.447, 0.034) | 0.09 | Non firearm homicide before law^1^ | -22.965% (-56.36%, 3.298%) |
| Additional trend in firearm homicide before law | 0.002 (-0.029, 0.033) | 0.91 | Firearm homicide before law^1^ | -22.741% (-55.026%, 2.820%) |
| Change in trend in non firearm homicide after law | -0.020 (-0.119, 0.079) | 0.69 | Non firearm homicide after law^1^ | -25.461% (-52.348%, -3.319%) |
| Additional change in trend of firearm homicide after law | -0.017 (-0.052, 0.018) | 0.34 | Firearm homicide after law^1^ | -27.357% (-55.051%, -4.611%) |
| Rate ratio of mortality at start | -0.871 (-1.059, -0.682) | <0.01 | Rate ratio of mortality at start | -138.888% (-188.409%, -97.869%) |
| Non firearm level effect | 0.400 (-0.952, 1.752) | 0.56 | Non firearm level effect | 32.974% (-159.119%, 82.662%) |
| Firearm level effect | 0.372 (-1.052, 1.796) | 0.61 | Firearm level effect | 31.076% (-186.364%, 83.411%) |
|  |  |  |  |  |
| **2001 Licensing** |  |  |  |  |
|  |  |  |  |  |
| Trend in non firearm homicide before law | -0.231 (-0.456, -0.006) | 0.04 | Non firearm homicide before law^1^ | -25.988% (-57.781%, -0.601%) |
| Additional trend in firearm homicide before law | -0.015 (-0.029, -0.002) | 0.03 | Firearm homicide before law^1^ | -27.914% (-59.92%, -2.314%) |
| Change in trend in non firearm homicide after law | 0.059 (-0.079, 0.197) | 0.40 | Non firearm homicide after law^1^ | -18.753% (-62.673%, 13.310%) |
| Additional change in trend of firearm homicide after law | 0.016 (-0.014, 0.047) | 0.29 | Firearm homicide after law^1^ | -18.599% (-62.855%, 13.630%) |
| Rate ratio of mortality at start | -0.779 (-0.925, -0.632) | <0.01 | Rate ratio of mortality at start | -117.842% (-152.16%, -88.195%) |
| Non firearm level effect | -1.225 (-3.972, 1.521) | 0.38 | Non firearm level effect | -240.474% (-5207.675%, 78.159%) |
| Firearm level effect | -1.843 (-4.58, 0.894) | 0.19 | Firearm level effect | -531.438% (-9647.452%, 59.096%) |
|  |  |  |  |  |

^1^Percent change per year
